# Pharmaceutical Utilization Review: A Five-Year Time Trend Analysis of the Pharmaceutical Market in Iran

**DOI:** 10.5812/ijpr-158927

**Published:** 2026-02-14

**Authors:** Mohammadamir Nemati, Behzad Fatemi, Fatemeh Soleymani, Meysam Seyedifar, Donya Zali, Zahra Shahali, Hossein Ranjbaran

**Affiliations:** 1 Department of Pharmacoeconomics and Pharmaceutical Administration, Faculty of Pharmacy, Tehran University of Medical Sciences (TUMS), Tehran, Iran; 2 Pharmaceutical Management and Economic Research Center, Institute of Pharmaceutical Sciences, Tehran University of Medical Sciences, Tehran, Iran; 3 National Center for Health Insurance Research, Tehran, Iran; 4 Immunogenetics Research Center, Faculty of Medicine, Mazandaran University of Medical Sciences, Sari, Iran

**Keywords:** Co-payment Policies, Drug Usage, Drug Utility Review, Pharmaceutical Policy, Pharmacoepidemiology, Prescription, Rational Use of Drugs

## Abstract

**Background and Objectives:**

The primary objective of this study was to compare the total and insurance-covered utilization of pharmaceuticals in Iran from March 2018 to March 2023. The findings of this study can offer valuable insights to healthcare professionals, researchers, and policymakers to make informed decisions.

**Methods:**

A retrospective cross-sectional study in Iran examined drug utilization patterns (specific databases, e.g., Iranian Health Insurance Organization and Social Security Organization) using Joinpoint regression. The study calculated the Defined Daily Doses per 1000 inhabitants per day (DID) for each medicine based on the anatomical therapeutic chemical/defined daily dose (ATC/DDD) system. All medicines with valid ATC/DDD assignments available during 2018 - 2023 were included to ensure comprehensive national coverage rather than a selected sample. Joinpoint regression was employed for trend analysis, focusing on annual and monthly percent changes in pharmaceutical utilization. Statistical analysis was conducted using MS Excel and Joinpoint software, with significance set at P-value < 0.05.

**Results:**

The study assessed 1185 ATC codes, out of which 751 were found eligible for analysis. Among these, 728 were covered by two major Iranian insurance organizations. The study found that the share of insurance utilization of these pharmaceuticals ranged from 27 to 48 percent. The trend utilization showed significant increases in overall utilization in classes C, J, L, N, and P. The utilization trends under insurance coverage revealed that classes L, R, and S showed a significant increase. After the implementation of the Daruyar Plan and the removal of the preferred currency exchange rate for all classes, there has been an increase in utilization under insurance coverage.

**Conclusions:**

This study reveals notable patterns and discrepancies in utilization levels within different drug classes, as well as the impact of insurance coverage and the Daruyar Plan on medication utilization.

## 1. Background

The pharmaceutical market has become increasingly dynamic and diverse, offering a wide range of effective medications to address various health conditions (1, 2). However, despite the availability of these treatments, there is often a misalignment between prescription practices and patient needs, leading to concerns about both over-prescription and under-prescription (1, 2). Prescription practices do not always align with the specific needs of the patient, and conversely, not all patient needs are addressed through pharmacotherapy. This situation has led to concerns about both over-prescription, which can be inappropriate and costly, and under-prescription (1, 2). Over-prescription can result in unnecessary costs and potential harm to patients, while under-prescription may leave critical health needs unaddressed. This discrepancy underscores the importance of optimizing drug utilization to ensure that medications are used rationally and effectively (3).

Rational drug use is a cornerstone of effective healthcare systems, and understanding the dynamics of the pharmaceutical market is essential for achieving this goal (3). Drug utilization research plays a critical role in evaluating patient exposure to medications over time, assessing the appropriateness of pharmaceutical use, and identifying trends in drug consumption. By analyzing epidemiological data, researchers can determine whether medications are being used excessively, inadequately, or in alignment with recommended guidelines (3).

Drug utilization evaluation (DUE) is a structured, ongoing process designed to ensure the appropriate use of medications based on specific criteria (3). Originating in the 1960s, DUE studies examine various aspects of drug marketing, distribution, prescribing, and usage within communities, with the aim of evaluating their medical, social, and economic implications (4). The primary objective of DUE is to promote rational drug therapy by ensuring that medications are prescribed based on thorough research, at the correct dosage, for the appropriate indication, and at a reasonable cost (5).

To facilitate drug utilization studies, the World Health Organization (WHO) developed the anatomical therapeutic chemical (ATC) Classification and defined daily dose (DDD) system. This globally recognized tool assigns each drug a unique code, enabling comparisons of drug usage at international, national, and regional levels (6, 7). The ATC/DDD methodology not only promotes the rational use of medications but also supports the availability of essential drugs, particularly in low-income countries (8, 9).

One key metric derived from this system is the DDD per 1000 inhabitants per day (DID), which is widely used to measure and compare pharmaceutical consumption across different regions. The DID index helps identify underutilization or overutilization of medications and evaluates the impact of pharmaceutical policies (10).

National pharmaceutical policies, which are integral to national health sector strategies, play a crucial role in ensuring public access to safe and effective medications. These policies provide health policymakers with a range of options to address the growing demand for advanced and often expensive drugs, which has led to increased healthcare expenditures in recent years (11, 12). In Iran, the pharmaceutical market has experienced significant growth, driven by both domestic production and imports (13, 14). However, policymakers face the challenge of selecting and implementing policies that balance the needs of the public with the economic realities of the pharmaceutical sector. Regular review and adjustment of these policies are necessary to respond to internal and external factors influencing drug availability and affordability (15).

Recent pharmaceutical policies in Iran, such as the Daruyar Policy and the Foreign Currencies Exchange Rate Policy, aim to address these challenges. The Daruyar Policy, approved in 2022, directs drug subsidies to the final consumer, with the goal of increasing the number of drugs covered by insurance while reducing out-of-pocket costs for patients. The Foreign Currencies Exchange Rate Policy, implemented in 2002 and refined in 2022, seeks to create a fair and transparent currency exchange system within the pharmaceutical industry. These policies reflect ongoing efforts to improve drug accessibility and affordability in Iran.

Despite these efforts, there is a lack of comprehensive studies evaluating the impact of these policies on pharmaceutical utilization trends in Iran. Furthermore, there is limited research on how insurance coverage influences drug consumption patterns in the country. 

## 2. Objectives

This study aims to fill these gaps by evaluating pharmaceutical utilization trends in Iran from 2018 to 2022, with a focus on the impact of insurance coverage. Using the WHO ATC/DDD classification system, this research analyzed previously recorded data to provide insights into drug consumption patterns. The findings will enhance the informed decision-making process for policymakers, physicians, pharmacists, and researchers, ultimately contributing to improved health-related quality of life (HRQOL) for patients.

In addition to policy and utilization perspectives, ethical considerations in pharmaceutical market management are increasingly emphasized. Recent systematic reviews highlight strategies for managing conflicts of interest in the pharmaceutical sector, underscoring the importance of transparency and accountability in utilization analyses (16). Moreover, national health trends, such as the five-year trajectory of gastric cancer in Northern Iran, provide important epidemiological context that may correlate with observed pharmaceutical consumption patterns (17). These studies complement our investigation by situating pharmaceutical utilization within broader health system and disease burden dynamics.

## 3. Methods

### 3.1. Study Design

A cross-sectional study was conducted to analyze the trends of DID medical utilization for every pharmaceutical ATC group from March 21, 2018 to March 20, 2023 in Iran, and this article was written based on the reporting of studies conducted using Observational Routinely Collected Health Data for Pharmacoepidemiology (RECORD-PE) checklist (18). The official reports of pharmaceutical statistics of Iran by the Food and Drug Administration were used to check the utilization of pharmaceuticals. Moreover, to compare pharmaceutical usage and insurance coverage in Iran, we collected data from two major Iranian insurance organizations: the National Center for Health Insurance Research and Tamin Ejtemaei Insurance. It is important to note that our study did not include data from the armed forces insurance, which covers more than 4 million people. This limitation is further discussed in the limitations section of the study.

Eligibility criteria were defined to ensure consistency with WHO ATC/DDD methodology. All medicines with valid ATC codes and WHO-assigned DDDs available between 2018 - 2023 were included. However, some medicines were excluded from our study based on the following exclusion criteria:

(1) Medicines that are not assigned an ATC code on the WHO website.

(2) Medicines for which DDD is not assigned on the WHO website [most topical products of class D, serums (sub-class B05), vaccines (sub-class J07), antineoplastic agents from sub-class L01, local and systemic anesthesia (sub-class N01), eye and ear drugs (most drugs of class S), and contrast agents (sub-class V08)].

(3) Combination drugs whose equivalents were not available on the WHO website.

(4) Medicines specifically intended for pediatric use.

### 3.2. Statistical Analysis

All pharmaceutical utilizations, in each time (month or year) of the study period, were converted to the DID indicator according to the following formula (10):


$$DDD\;per\;1000\;persons\;per\;day=\frac{N\times M\times Q\times 1000}{DDD\times P\times T}$$

Where: N refers to the number of drugs utilized; M refers to the strength of the dosage form; Q refers to the package size; P, population, reflected sample size; and T study period (days).

Between 2018 and 2022, the number of Iranian adults (aged 12 and above) who were covered by the two main Iranian insurance organizations ranged from 52 to 56 million people. This means that about 77% of the Iranian population was under these two main insurance organizations.

To evaluate the average annual percent change (AAPC) of pharmaceutical utilization, both total and under insurance, the Joinpoint regression method was adopted. Additionally, the impact of two major national drug policies, changes in disease prevalences, and global phenomena, such as COVID-19, on pharmaceutical utilization were analyzed.

Initially, to review the trend of utilization based on DID, we checked the total utilization at the 1st ATC level. Then, estimated DID values were compared with utilization under insurance at the 1st level of the ATC code. In cases where inconsistencies were observed in DID trends at ATC Level 1, further investigations were conducted at other ATC levels to identify specific drug groups or therapeutic categories contributing to these variations. This approach helped determine the underlying reasons for discrepancies between DID trends in insurance-covered drugs and total drug utilization.

Trends in pharmaceutical utilization were analyzed using Joinpoint regression (Joinpoint Software version 5.0.2, National Cancer Institute) to identify significant changes in annual percent change (APC) and monthly percent change (MPC). Statistical significance was set at P < 0.05. To address potential confounding:

Stratified analysis: Trends were evaluated separately for each ATC therapeutic class to control for class-specific prescribing patterns.

Policy and pandemic adjustments: Sensitivity analyses included covariates for the Daruyar Plan (June 2022) and COVID-19 phases (pre-pandemic, pandemic, post-pandemic).

Data Segmentation: The study period was divided into three phases (2018 - 2019, 2020 - 2021, 2022 - 2023) to account for temporal disruptions.

## 4. Results

### 4.1. Descriptive Analysis

A total of 1185 ATC codes were evaluated between 2018 and 2023, representing distinct drug molecules, each of which may include various dosage forms (e.g., tablets, capsules, syrups). Based on our eligibility criteria, we selected 751 ATC codes for the final analysis. Out of all the medicines included, 728 were covered by two major Iranian insurance organizations (Appendix 1 in Supplementary File).

**Figure 1.**
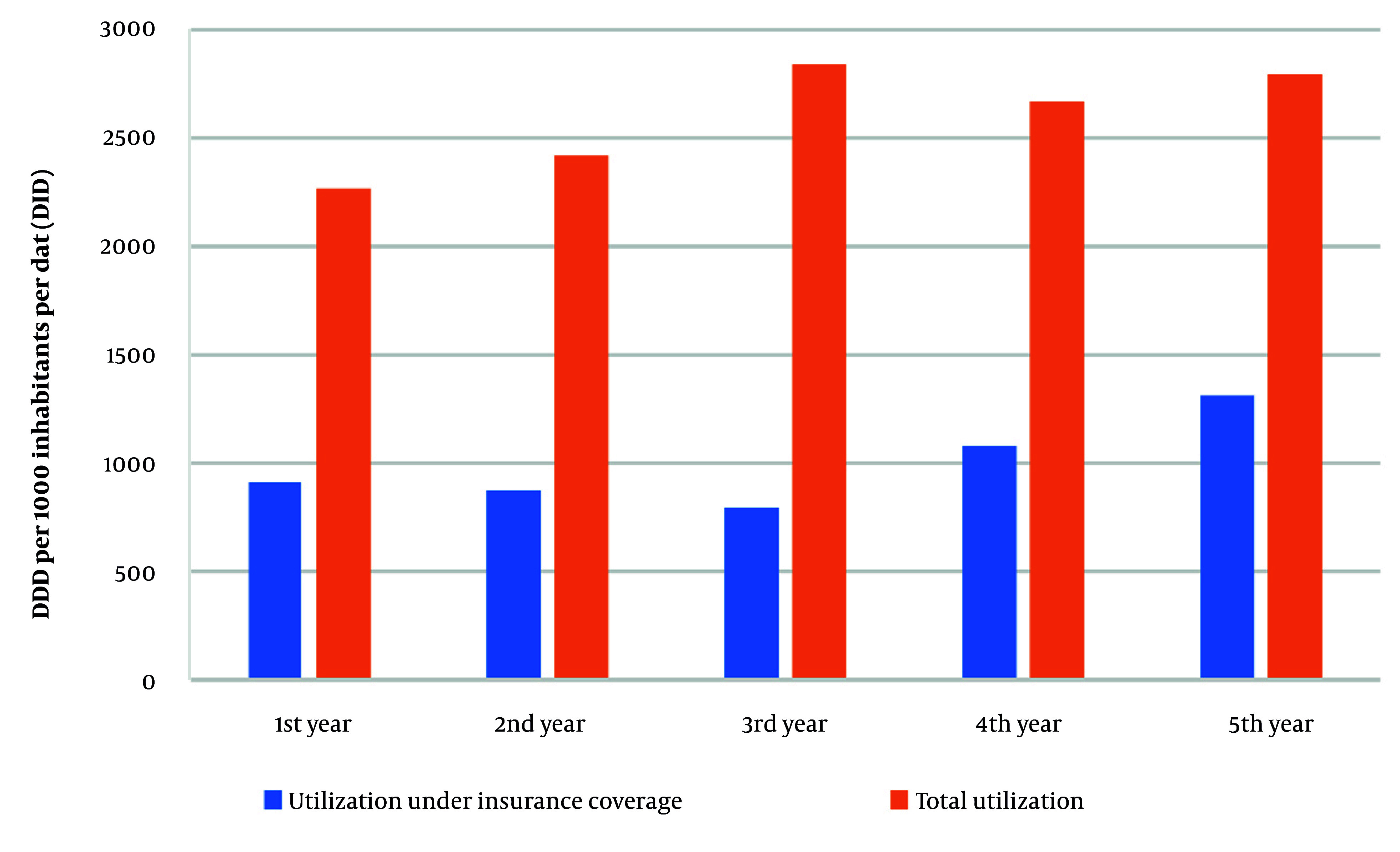
The percent of utilization under insurance based on DID. DDD, defined daily dose; DID, DDD per 1000 inhabitants per day

The calculated DID showed that the share of the two major Iranian insurance organizations was between 27 to 48 percent of the annual utilization (Figure 1). 

In addition, class A had the highest percent of DID in the 1st ATC level (Appendix 1 in Supplementary File).

### 4.2. Statistical Analysis

#### 4.2.1. DID Utilization Annual Percentage Change 

Based on the Annual Percentage Change (APC) Index in Table 1, the utilization trends in classes C (cardiovascular system), J (anti-infectives for systemic use), L (antineoplastic and immunomodulating agents), N (nervous system), and P (antiparasitic products, insecticides and repellents) have been significantly increasing from March 2018 to March 2023, and the utilization trend in class A has been significantly increasing from March 2018 to March 2021. However, since then, it has been declining (Appendix 2-13 in Supplementary File).

**Table 1.** Annual Percent Change

1^st^ Level (Joinpoints)/Segment	Lower Endpoint-Upper Endpoint (March)	APC (95% CI)
**A – 1 **		
1	2018 - 2021	9.7783 ^a^ (2.40 - 18.16)
2	2021 - 2023	-8.5221^ a^ (-15.06 - -1.95)
**B - 1**		
1	2018 - 2021	7.6003 ^a^ (4.80 - 10.70)
2	2021 - 2023	14.6414 ^a^ (11.35 - 17.75)
**C - 0**		
1	2018 - 2023	13.5999 ^a^ (9.75 - 17.48)
**G - 1**		
1	2018 - 2021	2.1175 (-6.52 - 12.87)
2	2021 - 2023	17.6718 ^a^ (6.46 - 28.95)
**H - 1**		
1	2018 - 2021	19.5312 ^a^ (1.22 - 42.83)
2	2021 - 2023	-0.3118 (-16.96 - 17.96)
**J - 0**		
1	2018 - 2023	9.2872 ^a^ (1.38 - 17.61)
**L - 0**		
1	2018 - 2023	11.2070 ^a^ (3.40 - 19.26)
**M - 1**		
1	2018 - 2021	5.7806 ^a^ (3.06 - 8.79)
2	2021 - 2023	15.7122 ^a^ (12.47 - 18.82)
**N - 0**		
1	2018 - 2023	18.0618 ^a^ (11.42 - 24.87)
**P - 0**		
1	2018 - 2023	7.9453 ^a^ (3.08 - 12.92)
**R - 1**		
1	2018 - 2021	6.2682 ^a^ (4.83 - 7.82)
2	2021 - 2023	39.3253^a^ (37.32 - 41.27)
**V - 1**		
1	2018 - 2021	2.8200 (-4.35 - 11.05)
2	2021 - 2023	22.3628 ^a^ (13.25 - 31.74)

Abbreviation: APC, annual percent change; CI, confidence interval; A, alimentary tract and metabolism; B, blood and blood forming organs; C, cardiovascular system; G, genito urinary system and sex hormones; H, systemic hormonal preparations, Excl. sex hormones and insulins; J, anti-infectives for systemic use; L, antineoplastic and immunomodulating agents; M, musculo-skeletal system; N, nervous system; P, antiparasitic products, insecticides and repellents; R, respiratory system; V, various.

^a^ Statistically significant.

As shown in Table 1, the highest AAPC was for classes R [respiratory system (21.68%)], N [nervous system (18.06%)], and C [cardiovascular system (13.59%)], respectively.

**Figure 2.**
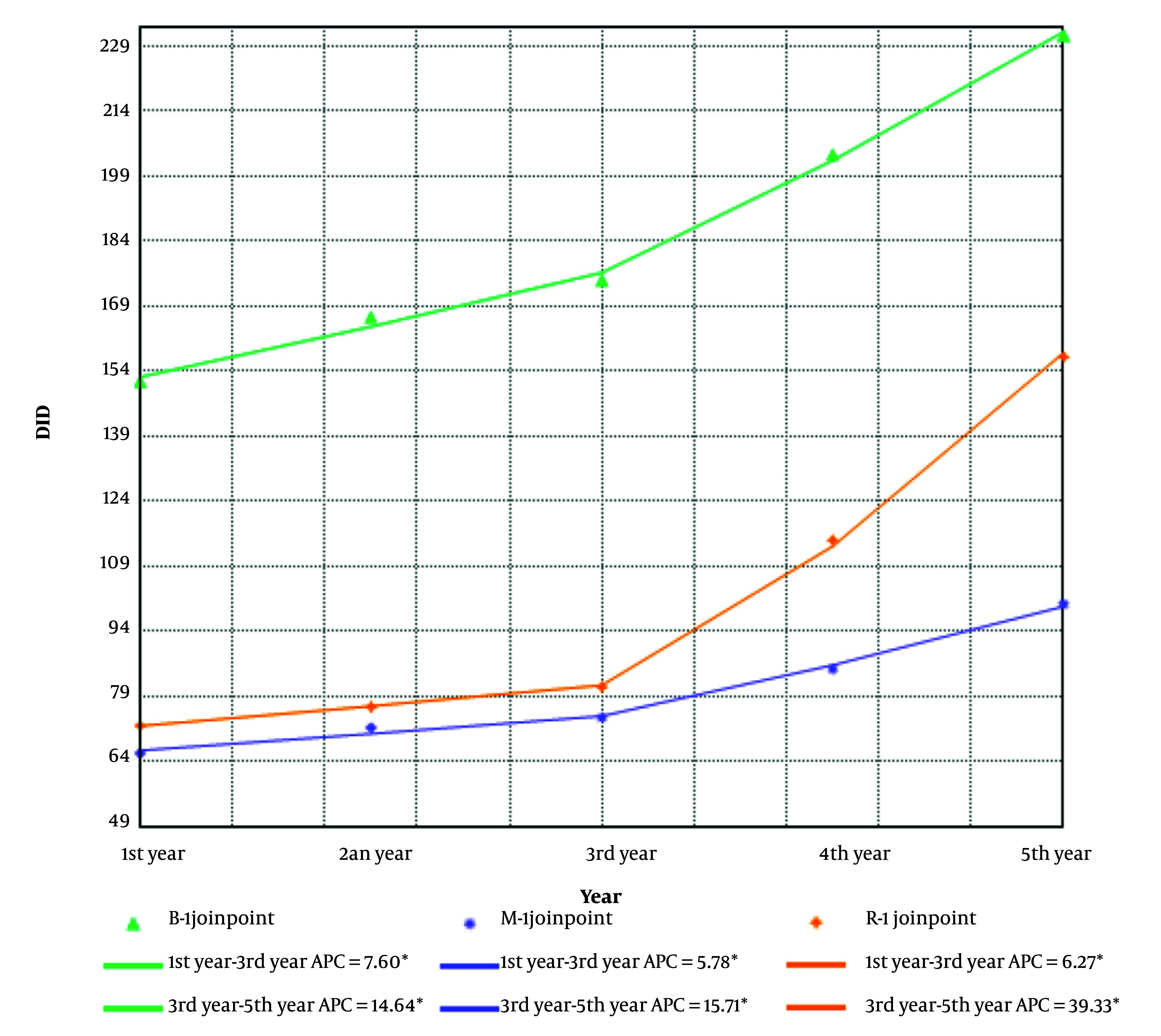
Trend total utilization of classes B, M, R. DDD, defined daily dose; DID, DDD per 1000 inhabitants per day

Based on APC, there is no statistically significant difference in the utilization trends of classes D (dermatological) and S (sensory organs) from March 2018 to March 2023. Also, the trend of drug utilization in classes B (blood and blood forming organs), M (musculo-skeletal system), and R (respiratory system) were significantly increasing from March 2018 to March 2023, but this trend increases more steeply from March 2021 to March 2023 in class R (Figure 2). 

#### 4.2.2. Joinpoint Regression Analysis of Pharmaceutical DID Utilization Under Insurance Coverage from March 2018 to March 2023

Based on the MPC Index in Table 2, the utilization trends in classes L, R, and S have been significantly increasing from March 2018 to March 2023, but the utilization trend in class R from December 2019 to March 2020 has been declining (Appendix 14-24 in Supplementary File).

**Table 2.** Monthly Percent Change

1^st^ Level (Joinpoints)/Segment	Lower Endpoint - Upper Endpoint	APC (95% CI)
**B - 1 **		
1	Mar. 2018 - 2021	-0.0510 (-1.37 to 0.46)
2	Mar. 2021 - Feb. 2023	2.0604 ^a^ (1.02 to 5.81)
**C - 1**		
1	Mar. 2018 - 2022	-0.1616 (-0.48 to 0.11)
2	Mar. 2022 - Feb. 2023	5.4118 ^a^ (2.91 to 10.85)
**H - 2**		
1	Mar. 2018 - Dec. 2019	0.9515 (-0.07 to 2.90)
2	Dec. 2019 - Mar. 2020	-13.4855 ^a^ (-17.57 to -0.96)
3	Mar. 2020 - Feb. 2023	3.3976 ^a^ (2.87 to 4.05)
**J - 4**		
1	Mar. 2018 - Dec. 2018	3.3521 (-0.85 to 19.62)
2	Dec. 2018 - Feb. 2021	-1.8236 (-10.92 to 8.37)
3	Feb. 2021 - Jan. 2022	9.0973 (-3.34 to 24.07)
4	Jan. 2022 - Apr. 2022	-15.1579 (-21.50 to 6.74)
5	Apr. 2022 - Feb. 2023	10.5979 ^a^ (5.85 to 20.49)
**L - 0**		
1	Mar. 2018 - Feb. 2023	0.7304 ^a^ (0.54 to 0.91)
**M - 1**		
1	Mar. 2018 - Nov. 2020	-0.5459 ^a^ (-1.20 to -0.01)
2	Nov. 2020 - Feb. 2023	4.1934 ^a^ (3.46 to 5.10)
**N - 1**		
1	Mar. 2018 - Feb. 2021	-0.1845 (-0.75 to 0.25)
2	Feb. 2021 - Feb. 2023	3.0816 ^a^ (2.29 to 4.28)
**R - 3**		
1	Mar. 2018 - Dec. 2019	2.2782 ^a^ (0.61 to 4.62)
2	Dec. 2019 - Mar. 2020	-22.0206 (-27.32 to 0.73)
4	May 2022 - Feb. 2023	12.8289 ^a^ (6.07 to 33.74)
**S - 2**		
1	Mar. 2018 - Apr. 2019	0.5297 (-12.94 to 5.33)
2	Apr. 2019 - Jul. 2019	47.0855 ^a^ (10.19 to 63.06)
3	Jul. 2019 - Feb. 2023	0.8655 (-0.003 to 1.52)

Abbreviations: MPC, monthly percent change; CI, confidence interval; B, blood and blood forming organs; C, cardiovascular system; H, systemic hormonal preparations, Excl. sex hormones and insulins; J, anti-infectives for systemic use; L, antineoplastic and immunomodulating agents; M, musculo-skeletal system; N, nervous system; P, R, respiratory system; S, sensory organs.

^a^ Statistically significant.

As shown in Table 2, the highest average monthly percent change (AMPC) was for classes R (3.16%), S (2.74%), H (1.59%), and M (1.59%), respectively.

The survey was conducted in insurances during 60 months from March 2018 to March 2023. The utilization under insurance coverage trend for classes A and D showed a decline from March 2018 to March 2021. However, since then, it has been on the rise with two distinct slopes (Appendix 14, 17 in Supplementary File). The utilization trend for classes B and C showed a decline from March 2018 to March 2021. However, since then, it has been on the rise (Appendix 20, 21 in Supplementary File). The trend of utilization under insurance coverage in classes G and H has been on the rise with two distinct slopes (Appendix 18, 19 in Supplementary File), and showed a declining trend only in March 2019.

The utilization under insurance coverage trend of class L has consistently been upward. The trend of utilization in classes M, N, and P showed a decline from the beginning until March 2020, and since then, it has been on the rise. The trend of utilization for class V showed an increase with two distinct slopes, but a sharp drop occurred from September 2020 to March 2021.

**Figure 3.**
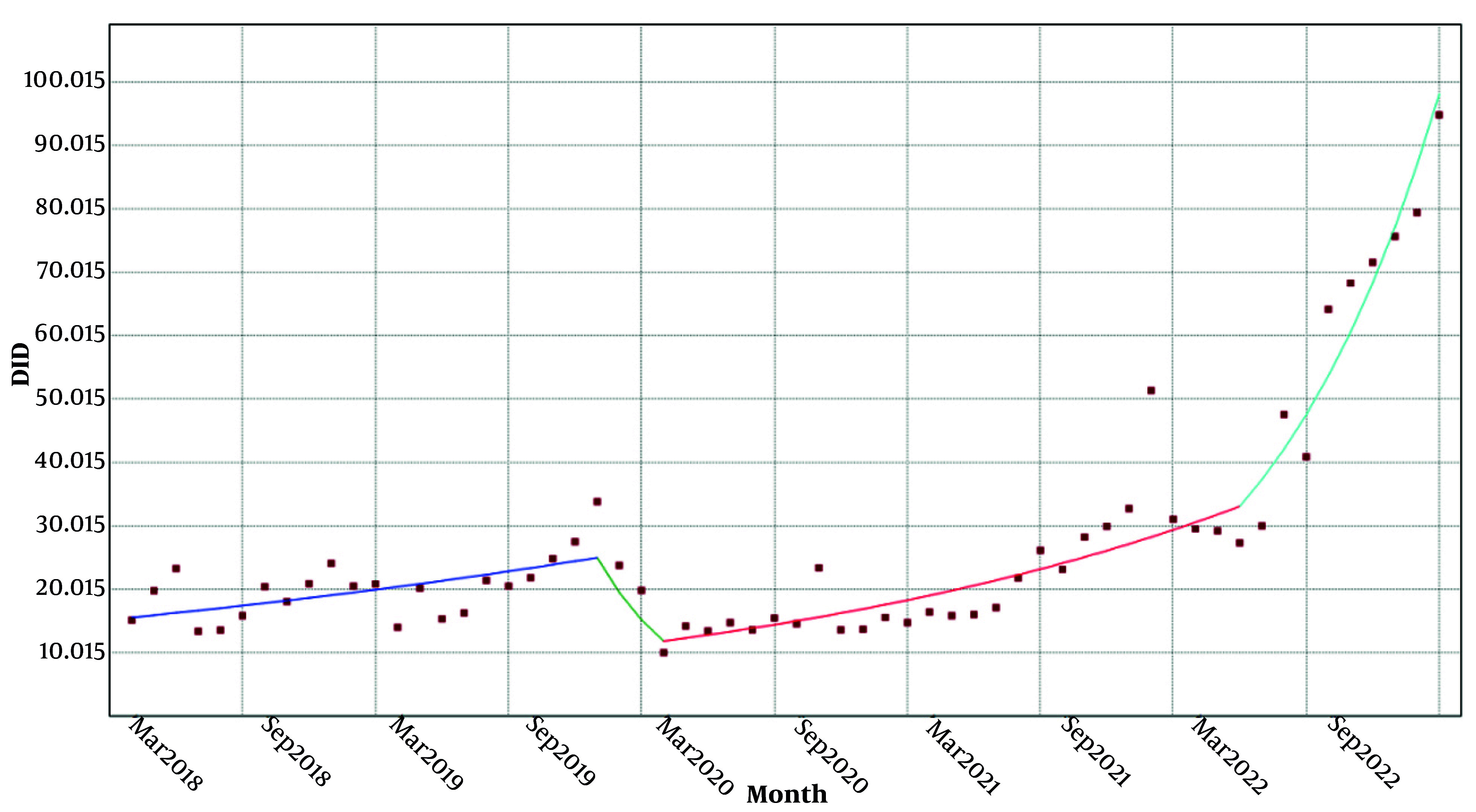
Trend of insurance utilization in class J. DDD, defined daily dose; DID, DDD per 1000 inhabitants per day; J, anti-infectives for Systemic Use

The utilization under insurance coverage trend of class J during 2018 showed an increase. However, since then, it has been on the decline from March 2019 to March 2020. However, since then, it has been on the rise with two distinct slopes and only a decrease occurred from February to May 2022 (Figure 3). 

**Figure 4.**
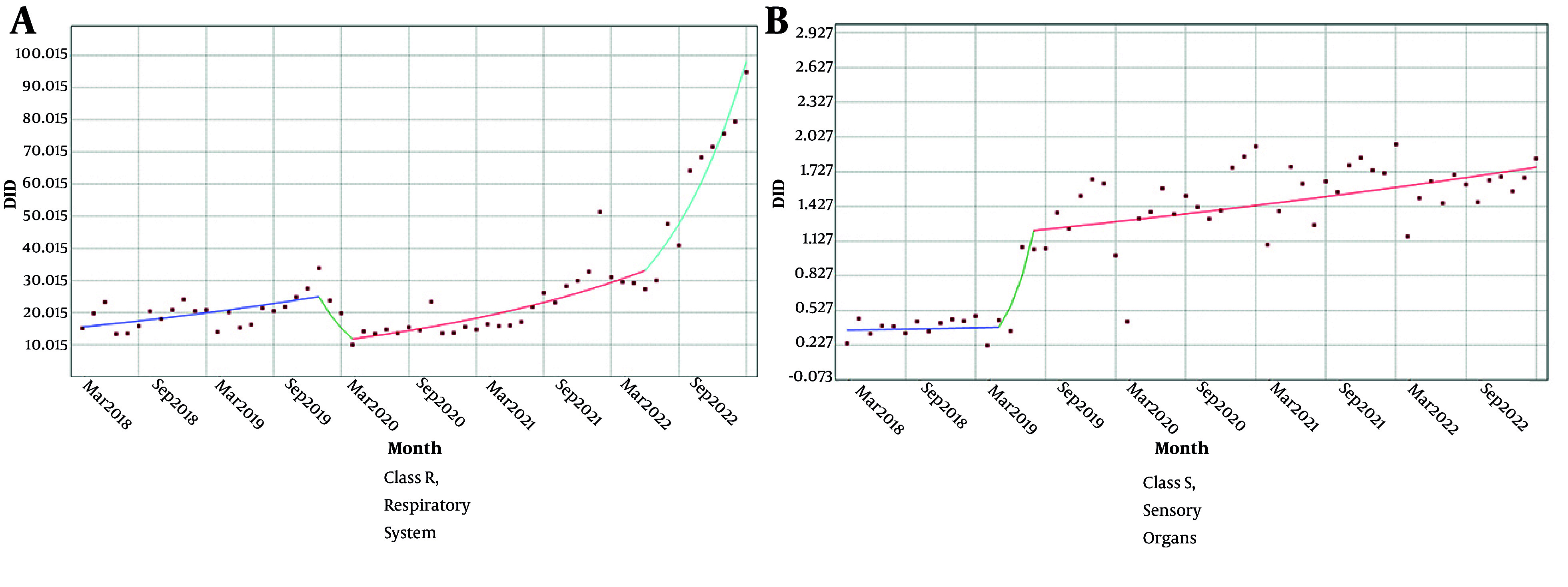
Trend of utilization under insurance coverage in A, class R; and B, class S; DDD per 1000 inhabitants per day (abbreviations: R, respiratory system; S, sensory organs)

The trend of utilization under insurance coverage in classes R and S has been on the rise with three distinct slopes, and it only declined in March 2020 in class R (Figure 4). 

### 4.3. The Impact of the Daruyar Policy and the Implementation of a Single Exchange Rate for USD to IRRial on Pharmaceutical Utilization in Iran

The Daruyar policy started in June 2022, and the implementation of the single exchange rate of currencies was in March 2022. At the specified times for the start of the Daruyar policy and the implementation of the single exchange rate of currencies in all classes, there has been a rise in total and utilization under insurance coverage. However, in classes A, H, L, and S, there was a decline in total utilization.

### 4.4. Comparing Total and Covered Utilizations in 5th ATC Level

Also, based on the highest amount of DID between total utilization and utilization under insurance coverage data, a more detailed investigation was performed. The highest amount of DID between total utilization and utilization under insurance coverage utilization data was observed in drugs such as Acetylsalicylic acid (B01AC06), Atorvastatin (C01AA05), Metformin (A10BA02), Losartan (C09CA01), Famotidine (A02BA03), Colecalciferol (A11CC05), Thiamine (A11DA01), and Diclofenac (M01AB05).

This review at the 5th ATC level indicated that there were many similarities in most DID of utilization under insurance coverage and total utilized medicines.

## 5. Discussion

This study delves into pharmaceutical utilization trends in Iran, focusing on 751 ATC codes, of which 728 were covered by the nation's two primary insurance organizations. Over a comprehensive 5-year period, we observed notable patterns in pharmaceutical utilization, particularly within classes A, C, B, and N, as determined by the DID index. Utilization levels within the ATC classification were 1578, 248, 185, and 165, respectively. Among these, key drugs such as Acetylsalicylic acid (B01AC06), Atorvastatin (C01AA05), Metformin (A10BA02), Losartan (C09CA01), Famotidine (A02BA03), Cholecalciferol (A11CC05), Thiamine (A11DA01), and Diclofenac (M01AB05) exhibited the highest DDD values. Our findings align with existing research on global pharmaceutical trends, including studies on the Global Burden of Disease (GBD) (19), the global use of medicines 2023 (20), and pharmaceutical utilization profiles in various countries (21-25). These comparisons reveal both similarities and differences in utilization trends, providing valuable insights into Iran's pharmaceutical landscape within the broader international context.

Furthermore, our investigation reveals that pharmaceutical utilization under insurance coverage ranged from 27% to 48% of the total national pharmaceutical utilization. Notably, this coverage percentage does not align proportionally with the proportion of individuals covered by the two main insurance plans in Iran, which represents approximately 77% of the total Iranian population. This discrepancy prompts several inquiries: Were healthcare services equally accessible to all patients under insurance plans? What was the prevalence of over-the-counter (OTC) sales of prescription-required medicines? Is there any incentive for pharmaceutical smuggling due to government subsidies on pharmaceutical provision in Iran? Additionally, has the rate of medication usage under both major insurance coverage plans been equitable? Addressing these questions holds significant potential for elucidating the landscape of pharmaceuticals and insurance in Iran.

We find that, based on average percentage change, class R utilization is trending in the total Iranian pharmaceutical market in the study period. However, there was an inconsistency between total market and under-insurance coverage utilizations in Iran.

As mentioned above, the utilization of total and under-insurance coverage within class A was notably prominent. However, this utilization was primarily attributed to the utilization of vitamins (Sub-class A11), which confirms the findings of studies conducted in Iran (26) and Ireland (25).

Sub-class analysis of class J revealed that the highest utilization in this sub-class was associated with Amoxicillin (J01CA04), Cefixime (J01DD08), and Azithromycin (J01FA10). This pattern has been consistently shown in the findings of previous studies conducted in Iran (27), underscoring its persistence within the country's context.

Time trend analysis of all reviewed pharmaceuticals indicated that the utilization trend in class A has significantly increased from March 2018 to March 2021. After that, it has been declining until March 2023. This change in trends of utilization in class A could have been affected by the COVID-19 pandemic. The ascending slope of utilization within this pharmaceutical class aligns with utilization in other countries (20, 22) and the growth trend observed in gastrointestinal disease and diabetic disease (19) during the period from March 2018 to March 2021. Following the end of the COVID-19 pandemic, a notable decline in vitamin utilization resulted in a shift in the utilization pattern within this drug class (25). However, the MPC of class A indicated that utilization under insurance coverage was significantly increasing. The observed inconsistency may be attributable to the OTC sale of sub-classes like vitamins and gastrointestinal pharmaceuticals.

Total utilization in class D (Dermatological) did not follow a specific trend. Within this class, there have been numerous fluctuations, exhibiting alternating decreases and increases. Our finding confirmed the results of another study from Iran, in which a general upward trend in class D medical utilization in Iran was observed (28). However, we detected a decline during 2019-2020, which could be the impact of the COVID-19 pandemic. Also, our finding is consistent with medical utilization in class D in Denmark in the same period of time (21). In addition, it is noteworthy that a large number of drugs in class D are topical formulations and had no DDD values, which was an exclusion criterion for the present study.

Our finding shows that the trend of utilization in class D, under insurance coverage, had a similar pattern observed in prescription trends in other countries (23-25, 28). Furthermore, the volume of this utilization based on basic insurance coverage corresponds to the prevalence of diseases in this class (19).

Total utilization in class J has shown an upward trend. In addition, data on the global utilization of antibiotics, derived from the sales volume of 71 countries and examining four broad-spectrum antibiotics, i.e., cephalosporins (J01DB, J01DC, J01DD, J01DE), penicillins (J01CA), macrolides (J01FA), and tetracyclines (J01AA), showed a different trend of utilization in these four subclasses. Global data showed that prior to the onset of the COVID-19 pandemic, antibiotic utilization was on the rise, but from the beginning of 2020, a downward trend emerged, and it returned to the trend before the start of the pandemic again in 2022 (29). The observed trend in the national utilization of two broad-spectrum antibiotics, i.e., cephalosporins and penicillins, aligns with the global pattern. However, in two other antibiotics (macrolides and tetracycline), this trend was increasing.

As mentioned above, a disparity exists between the national utilization of class J in Iran and their global utilization. One significant factor that may account for this discrepancy is the arbitrary use of antibiotics in Iran, particularly during the COVID-19 pandemic period.

In class J, the pattern of antibiotic utilization under insurance coverage displayed a relatively ascending trajectory, diverging from both the global trend (23-25, 30, 31) and prevailing prevalence rates (19). The widespread misuse of antimicrobials is a common occurrence, representing a significant driving factor for the emergence of antimicrobial resistance (AMR). This phenomenon is pervasive across healthcare settings and communities alike, posing formidable challenges to effective infection management and public health (32).

The total utilization trend within class R was aligned with global utilization data from IQVIA (20) and drug utilization within this class in Denmark (21). We find significant growth in this drug class. However, the trend continued to rise until March 2023, potentially attributable to the over-the-counter (OTC) sale of prescription-required medicines in Iran. Conversely, in worldwide statistics, the growth trend increased until 2021, with a subsequent decrease in drug utilization up to 2023 after the pandemic. The trend in utilization under insurance coverage within class R demonstrated an increasing trajectory; however, a decline occurred in 2020. This decline somewhat aligns with utilization trends observed in other countries (23, 24).

After the gradual elimination of subsidized currencies for the pharmaceutical market in Iran (initiation of the Daruyar policy in June 2022), it was anticipated that the trend of arbitrary total drug utilization would decrease due to price increases. However, this decrease in utilization was only observed in OTC sub-classes of A, H, L, and S.

The Daruyar Plan had several goals, including reorganizing the drug subsidy system to ensure efficient distribution of drugs to patients, preventing reverse drug smuggling, and safeguarding the pharmaceutical industry. Additionally, the plan aimed to increase insurance coverage for patients while reducing their out-of-pocket (OOP) expenditures. This way, patients could benefit from insurance coverage and avoid paying a significant amount of OOP expenses for their medications. The Daruyar Plan was implemented to simplify the drug subsidy system and benefit patients and the pharmaceutical industry. Our research shows that the Daruyar plan resulted in a significant increase in the utilization of insurance coverage for medications across all ATC classes. This trend may be attributed to more doctor visits and an increase in prescriptions for medications that were previously available without a prescription and classified as OTC sales.

This study employed Joinpoint regression as a descriptive method to identify changes in utilization trends and estimate APC/AAPC. By design, Joinpoint does not adjust for confounders and cannot establish causal relationships. Accordingly, our objective was not to quantify the causal effects of COVID‑19 or national policy reforms, but rather to describe overall national patterns. The breakpoints observed in our analysis should therefore be interpreted cautiously, as their timing does not consistently align with events such as the Daruyar Plan, the single exchange rate reform, or pandemic phases. These references were included only as contextual markers to aid interpretation. The observed shifts in utilization likely reflect multiple unmeasured influences, including drug shortages, procurement delays, pricing policies, insurance reimbursement changes, and broader supply‑chain instabilities. Consistent with this descriptive framework, the findings are generalizable only to the populations and data sources analyzed, and residual confounding and unmeasured policy‑level or market‑level disruptions remain important limitations.

Our findings should also be interpreted in light of broader developments in the Iranian health sector. For instance, recent evidence on gastric cancer trends in Northern Iran highlights how disease epidemiology can shape pharmaceutical demand and utilization trajectories (17). Similarly, systematic reviews on conflict-of-interest management in the pharmaceutical industry emphasize the need for ethical oversight and policy safeguards when interpreting market-level utilization data (16). Together, these complementary perspectives reinforce the relevance of our study within both the clinical and policy domains.

In summary, this study underscores the intricate interplay between pharmaceutical utilization, insurance coverage, and external factors. It emphasizes the necessity for comprehensive healthcare policies to address disparities and optimize resource allocation.

### 5.1. Limitations

Interpreting the findings of this study requires caution due to several reasons. Firstly, the ATC/DDD system has inherent constraints, particularly for pediatric medicines, topical agents, and drugs with variable real-world dosing, which may lead to inaccuracies when estimating utilization. Secondly, estimating drug usage for medications with multiple purposes can introduce further errors. Thirdly, the study acknowledges limitations associated with the Daruyar plan and emphasizes the importance of data after March 2023 for a comprehensive evaluation. Fourthly, the COVID-19 pandemic, which occurred during the study period, may have significantly influenced drug utilization patterns through changes in healthcare access, prescribing behaviors, and public health priorities. Fifthly, over-the-counter (OTC) sales and pharmaceutical smuggling, prevalent in Iran, could not be quantified, potentially underestimating total utilization. In addition, the timing of detected utilization breakpoints did not perfectly align with major policy changes, suggesting that unmeasured factors such as drug shortages, procurement delays, reimbursement adjustments, and supply-chain instabilities may have contributed to observed trends. Furthermore, the exclusion of Armed Forces Insurance, which covers a large beneficiary population, introduces potential selection bias that should be considered when generalizing results.

### 5.2. Conclusions

This study on pharmaceutical utilization trends in Iran reveals notable patterns and discrepancies in utilization levels within different drug classes, as well as the impact of insurance coverage and the Daruyar Plan on medication utilization. The findings highlight the need for further investigation into healthcare accessibility, over-the-counter sales, pharmaceutical smuggling, and equitable medication usage under insurance coverage in Iran.

ijpr-25-1-158927-s001.pdf

## Data Availability

The dataset presented in the study is available on request from the corresponding author during submission or after publication.
