# Supplementary material for: Pharmaceutical Utilization Review: A Five-Year Time Trend Analysis of the Pharmaceutical Market in Iran
Source: Iran J Pharm Res. 2026 Feb 14;25(1):e158927. doi: 10.5812/ijpr-158927 (PMC12933975; doi:10.5812/ijpr-158927)
Supplement: ijpr-25-1-158927-s001.pdf [file ijpr-25-1-158927-s001.pdf]

Appendix 1

ATC code	Generic Name	Coverage of Insurance
A01AA01	FLUORIDE SODIUM	Yes
A02AB01	ALUMINIUM HYDROXIDE	Yes
A02BA01	CIMETIDINE	Yes
A02BC01	OMEPRAZOLE	Yes
A03AD01	PAPAVERINE HYDROCHLORIDE	Yes
A03BA01	ATROPINE SULFATE	Yes
A03BB01	HYOSCINE-N-BUTYL BROMIDE	Yes
A03FA01	METOCLOPRAMIDE	Yes
A04AA01	ONDANSETRON	Yes
A07BA01	CHARCOAL ACTIVATED	Yes
A07DA01	DIPHENOXYLATE	Yes
A07EC01	SULFASALAZINE	Yes
A08AB01	ORLISTAT	Yes
A10BB01	GLIBENCLAMIDE	Yes
A10BF01	ACARBOSE	Yes
A10BH01	SITAGLIPTIN (AS PHOSPHATE)	Yes
A10BK01	DAPAGLIFLOZIN (AS PROPANEDIOL)	Yes
A11CA01	VITAMIN A	Yes
A11DA01	VITAMIN B1 (THIAMINE)	Yes
A11GA01	VITAMIN C	Yes
A12BA01	POTASSIUM CHLORIDE	Yes
A14AB01	NANDROLONE DECANOATE	Yes
A16AA01	L-CARNITINE	Yes
B01AB01	HEPARIN SODIUM	Yes
B01AF01	RIVAROXABAN	Yes
B02AB01	APROTININ	Yes
B02BA01	VITAMIN K1 (PHYTOMENADIONE)	Yes
B02BB01	fibrinogen	Yes
B03AE01	HEMATINIC	Yes

B03BA01	VITAMIN B12	Yes
B03BB01	FOLIC ACID	Yes
B03XA01	EPOETIN	Yes
C01BA01	QUINIDINE SULFATE	Yes
C01BB01	LIDOCAINE HYDROCHLORIDE	Yes
C01BD01	AMIODARONE HYDROCHLORIDE	Yes
C01EA01	PROSTAGLANDIN E1 (ALPROSTADIL)	Yes
C02AC01	CLONIDINE HYDROCHLORIDE	Yes
C02CA01	PRAZOSIN	Yes
C02DC01	MINOXIDIL	Yes
C02DD01	NITROPRUSSIDE SODIUM	Yes
C02KX01	BOSENTAN	Yes
C03AX01	TRIAMTERENE / HYDROCHLOROTHIAZIDE	Yes
C03CA01	FUROSEMIDE	Yes
C03DA01	SPIRONOLACTONE	Yes
C03EA01	AMILORIDE / HYROCHLOROTHIAZIDE	Yes
C04AA01	ISOXSUPRINE HYDROCHLORIDE	Yes
C07AG01	LABETALOL HYDROCHLORIDE	Yes
C08CA01	AMLODIPINE (AS BESILATE)	Yes
C08DA01	VERAPAMIL HYDROCHLORIDE	Yes
C08DB01	DILTIAZEM HYDROCHLORIDE	Yes
C09AA01	CAPTOPRIL	Yes
C09CA01	LOSARTAN POTASSIUM	Yes
C09DB01	AMLODIPINE (AS BESILATE) / VALSARTAN	Yes
C10AA01	SIMVASTATIN	Yes
C10AB01	CLOFIBRATE	Yes
C10AC01	CHOLESTYRAMINE	Yes
D10BA01	ISOTRETINOIN	Yes
G01AA01	NYSTATIN	Yes
G01AC01	IODOQUINOL	Yes
G01AF01	METRONIDAZOLE	Yes

G02CA01	RITODRINE	Yes
G02CB01	BROMOCRIPTINE (AS MESYLATE)	Yes
G03AD01	LEVONORGESTREL	Yes
G03CA01	ETHINYLESTRADIOL	Yes
G03DB01	DYDROGESTERONE	Yes
G03GA01	CHORIONIC GONADOTROPHIN (HUMAN)	Yes
G03HA01	CYPROTERONE ACETATE	Yes
G03XA01	DANAZOL	Yes
G03XC01	RALOXIFENE HYDROCHLORIDE	Yes
G04CB01	FINASTERIDE	Yes
H01AB01	THYROTROPIN ALFA	Yes
H01AC01	SOMATROPIN	Yes
H01CB01	SOMATOSTATIN	Yes
H01CC01	GANIRELIX	Yes
H02AB01	BETAMETHASONE (AS DISODIUM PHOSPHATE)	Yes
H03AA01	LEVOTHYROXINE SODIUM	Yes
H04AA01	GLUCAGON	Yes
H05BA01	CALCITONIN, SALMON	Yes
H05BX01	CINACALCET (AS HYDROCHLORIDE)	Yes
J01CA01	AMPICILLIN	Yes
J01CE01	PENICILLIN G POTASSIUM	Yes
J01CR01	AMPICILLIN / SULBACTAM	Yes
J01DB01	CEFALEXIN	Yes
J01DD01	CEFOTAXIME	Yes
J01DE01	CEFEPIME	Yes
J01EE01	CO-TRIMOXAZOLE	Yes
J01FA01	ERYTHROMYCIN	Yes
J01FF01	CLINDAMYCIN (AS HYDROCHLORIDE)	Yes
J01GA01	STREPTOMYCIN (AS SULFATE)	Yes
J01GB01	TOBRAMYCIN	Yes
J01MA01	OFLOXACIN	Yes

J01RA01	PENICILLIN 6-3-3	Yes
J01XA01	VANCOMYCIN (AS HYDROCHLORIDE)	Yes
J01XB01	COLISTIMETHATE SODIUM	Yes
J01XD01	METRONIDAZOLE	Yes
J01XE01	NITROFURANTOIN	Yes
J01XX01	FOSFOMYCIN (AS TROMETAMOL)	Yes
J02AA01	AMPHOTERICIN B	Yes
J02AC01	FLUCONAZOLE	Yes
J04AC01	ISONIAZID	Yes
J05AB01	ACICLOVIR	Yes
J05AP01	Ribavirin	Yes
L01EA01	IMATINIB (AS MESYLATE)	Yes
L01EB01	GEFITINIB	Yes
L01EF01	PALBOCICLIB	Yes
L01EL01	IBRUTINIB	Yes
L02AE01	BUSERELIN ACETATE	Yes
L02BA01	TAMOXIFEN	Yes
L02BB01	FLUTAMIDE	Yes
L04AB01	ETANERCEPT	Yes
L04AD01	CICLOSPORIN	Yes
L04AX01	AZATHIOPRINE	Yes
M01AB01	INDOMETHACIN	Yes
M01AC01	PIROXICAM	Yes
M01AE01	IBUPROFEN	Yes
M01AG01	MEFENAMIC ACID	Yes
M01AH01	CELECOXIB	Yes
M01CC01	PENICILLAMINE	Yes
M03BX01	BACLOFEN	Yes
M03CA01	DANTROLENE SODIUM	Yes
M04AA01	ALLOPURINOL	Yes
M04AC01	COLCHICINE	Yes

N02AA01	MORPHINE SULFATE	Yes
N02BE01	ACETAMINOPHEN	Yes
N02CA01	DIHYDROERGOTAMINE MESYLATE	Yes
N02CC01	SUMATRIPTAN (AS SUCCINATE)	Yes
N03AD01	ETHOSUXIMIDE	Yes
N03AE01	CLONAZEPAM	Yes
N03AF01	CARBAMAZEPINE	Yes
N03AG01	VALPROATE SODIUM	Yes
N04AA01	TRIHEXYPHENIDYL HYDROCHLORIDE	Yes
N04BB01	AMANTADINE HYDROCHLORIDE	Yes
N04BD01	SELEGILINE HYDROCHLORIDE	Yes
N05AA01	CHLORPROMAZINE HYDROCHLORIDE	Yes
N05AD01	HALOPERIDOL (AS DECANOATE)	Yes
N05AN01	LITHIUM CARBONATE	Yes
N05BA01	DIAZEPAM	Yes
N05BB01	HYDROXYZINE HYDROCHLORIDE	Yes
N05BE01	BUSPIRONE HYDROCHLORIDE	Yes
N05CD01	FLURAZEPAM	Yes
N06AA01	DESIPRAMINE HYDROCHLORIDE	Yes
N06AF01	ISOCARBOXAZID	Yes
N06BC01	CAFFEINE	Yes
N06DX01	MEMANTINE HYDROCHLORIDE	Yes
N07AA01	NEOSTIGMINE METHYLSULFATE	Yes
N07BC01	BUPRENORPHINE (AS HYDROCHLORIDE)	Yes
N07CA01	BETAHISTINE DIHYDROCHLORIDE	Yes
P01AB01	METRONIDAZOLE	Yes
P01BA01	CHLOROQUINE (AS PHOSPHATE)	Yes
P01BD01	PYRIMETHAMINE	Yes
P02CA01	MEBENDAZOLE	Yes
P02CB01	PIPERAZINE	Yes
P02CE01	LEVAMISOLE	Yes

P02CF01	IVERMECTIN	Yes
P02DA01	NICLOSAMIDE	Yes
R01AC01	CROMOLYN SODIUM	Yes
R03AL01	IPRATROPIUM BROMIDE / FENOTEROL HYDROBROMIDE	Yes
R03BA01	BECLOMETHASONE DIPROPIONATE	Yes
R03BC01	CROMOLYN SODIUM	Yes
R05CB01	ACETYLCYSTEINE	Yes
R07AB01	DOXAPRAM HYDROCHLORIDE	Yes
S01EC01	ACETAZOLAMIDE	Yes
S01ED01	TIMOLOL	Yes
A02AD01	ALUMINIUM HYDROXIDE / MAGNESIUM HYDROXIDE	Yes
A10AB01	INSULIN REGULAR	Yes
A10AC01	INSULIN ISOPHANE	Yes
A10AD01	INSULIN BIPHASIC LISPRO 25/75	Yes
A12CB01	ZINC	Yes
B01AD01	STREPTOKINASE	Yes
B02BA01	VITAMIN K1 (PHYTOMENADIONE)	Yes
B06AC01	C1-ESTRASE INHIBITOR PLASMA DERIVED INJECTION	Yes
C04AB01	PHENTOLAMINE MESYLATE	Yes
C04AC01	NICOTINIC ACID	Yes
C09DX01	AMLODIPINE (AS BESILATE) / VALSARTAN / HYDROCHLOROTHIAZIDE	Yes
D01BA01	GRISEOFULVIN	Yes
D10BA01	ISOTERTINOIN	Yes
G02AB01	METHYLERGONOVINE MALEATE	Yes
G03HB01	CYPROTERONE COMPOUND	Yes
H01BA01	VASOPRESSIN	Yes
J01GB01	TOBRAMYCIN (AS SULFATE)	Yes
J02AX01	FLUCYTOSINE	Yes
J05AF01	ZIDOVUDINE	Yes
J05AG01	NEVIRAPINE	Yes
M01AC01	PIROXICAM	Yes

M01AG01	MEFENAMIC ACID	No
N02AA01	MORPHINE SULFATE INJECTION PARENTERAL 10 mg/1mL	Yes
N02AD01	PENTAZOCINE (AS LACTATE)	Yes
N02BE01	acetaminophen	Yes
N05AF01	FLUPENTHIXOL DECANOATE	Yes
N07BB01	DISULFIRAM	Yes
P02BA01	PRAZIQUANTEL	Yes
R01AD01	BECLOMETHASONE DIPROPIONATE	Yes
R03DC01	ZAFIRLUKAST	Yes
V03AE01	SODIUM POLYSTYRENE SULFONATE	Yes
V08AD01	ETHIODIZED OIL	Yes
A02AF02	ALUMINIUM HYDROXIDE / MAGNESIUM HYDROXIDE / SIMETHICONE	Yes
A02BA02	RANITIDINE	Yes
A02BC02	PANTOPRAZOLE	Yes
A02BX02	SUCRALFATE	Yes
A05AA02	URSODEOXYCHOLIC ACID	Yes
A06AB02	BISACODYL	Yes
A07AA02	NYSTATIN	Yes
A07DA02	OPIUM	Yes
A07EC02	MESALAZINE	Yes
A09AA02	DIGESTIVE	Yes
A10BA02	METFORMIN HYDROCHLORIDE	Yes
A10BD02	METFORMIN HYDROCHLORIDE / GLIBENCLAMIDE	Yes
A10BJ02	LIRAGLUTIDE	Yes
A10BX02	REPAGLINIDE	Yes
A11HA02	VITAMIN B6	Yes
A12BA02	POTASSIUM CITRATE	Yes
A16AB02	IMIGLUCERASE	Yes
B01AD02	ALTEPLASE	Yes
B01AF02	APIXABAN	Yes
B02AA02	TRANEXAMIC ACID	Yes

B03AA02	IRON	Yes
C01BA02	PROCAINAMIDE HYDROCHLORIDE	Yes
C01BB02	MEXILETINE	Yes
C01CE02	MILRINONE LACTATE	Yes
C01DA02	NITROGLYCERIN	Yes
C02AB02	METHYLDOPA	Yes
C02DB02	HYDRALAZINE HYDROCHLORIDE	Yes
C04AX02	PHENOXYBENZAMINE	Yes
C07AB02	METOPROLOL TARTRATE	Yes
C07AG02	CARVEDILOL	Yes
C09AA02	ENALAPRIL MALEATE	Yes
C09CA02	EPROSARTAN (AS MESYLATE)	Yes
C10AA02	LOVASTATIN	Yes
D01BA02	TERBINAFINE HYDROCHLORIDE	Yes
D05BB02	ACITRETIN	Yes
D05BA02	METHOXSALEN	Yes
G01AF02	CLOTRIMAZOLE	Yes
G03BA02	METHYLTESTOSTERONE	Yes
G03DA02	MEDROXYPROGESTERONE ACETATE	Yes
G03DB02	MEGESTROL ACETATE	Yes
G03GA02	HUMAN PAPILLOMAVIRUS	Yes
G04CA02	TAMSULOSIN	Yes
G04CB02	DUTASTERIDE	Yes
H01AA02	TETRACOSACTIDE ACETATE	Yes
H01BA02	DESMOPRESSIN (AS ACETATE)	Yes
H01BB02	OXYTOCIN	Yes
H01CB02	OCTREOTIDE (AS ACETATE)	Yes
H01CC02	CETRORELIX	Yes
H02AA02	FLUDROCORTISONE ACETATE	Yes
H02AB02	DEXAMETHASONE	Yes
H03AA02	LIOTHYRONINE SODIUM	Yes

H03BA02	PROPYLTHIOURACIL	Yes
H05AA02	TERIPARATIDE	Yes
J01AA02	DOXYCYCLINE (AS MONOHYDRATE)	Yes
J01CE02	PENICILLIN V POTASSIUM	Yes
J01CF02	CLOXACILLIN (AS SODIUM)	Yes
J01CR02	CO-AMOXICLAV	Yes
J01DC02	CEFUROXIME	Yes
J01DD02	CEFTAZIDIME	Yes
J01DH02	MEROPENEM	Yes
J01EC02	SULFADIAZINE	Yes
J01FA02	SPIRAMYCIN	Yes
J01MA02	CIPROFLOXACIN	Yes
J01MB02	NALIDIXIC ACID	Yes
J01XA02	TEICOPLANIN	Yes
J02AB02	KETOCONAZOLE	Yes
J02AC02	ITRACONAZOLE	Yes
J04AB02	RIFAMPICIN	Yes
J04BA02	DAPSONE	Yes
J05AA02	DOXYCYCLINE (AS HYCLATE)	Yes
J05AH02	OSELTAMIVIR (AS PHOSPHATE)	Yes
L01EA02	DASATINIB	Yes
L01EB02	ERLOTINIB (AS HYDROCHLORIDE)	Yes
L01EX02	SORAFENIB (AS TOSYLATE)	Yes
L02AE02	LEUPRORELIN ACETATE	Yes
L03AA02	FILGRASTIM	Yes
L04AD02	TACROLIMUS	Yes
L04AX02	THALIDOMIDE	Yes
M01AE02	NAPROXEN	Yes
M03BX02	TIZANIDINE	Yes
N02AB02	PETHIDINE HYDROCHLORIDE	Yes
N02AX02	TRAMADOL HYDROCHLORIDE	Yes

N03AA02	PHENOBARBITAL SODIUM	Yes
N03AB02	PHENYTOIN SODIUM	Yes
N03AF02	OXANDROLONE	Yes
N04AA02	BIPERIDEN HYDROCHLORIDE	Yes
N04BA02	LEVODOPA / BENSERAZIDE	Yes
N05AB02	FLUPHENAZINE	Yes
N05AC02	THIORIDAZINE HYDROCHLORIDE	Yes
N05AG02	PIMOZIDE	Yes
N05AH02	CLOZAPINE	Yes
N05BA02	CHLORDIAZEPOXIDE	Yes
N05CD02	NITRAZEPAM	Yes
N05CF02	ZOLPIDEM	Yes
N06AA02	IMIPRAMINE HYDROCHLORIDE	Yes
N06AG02	MOCLOBEMIDE	Yes
N06DA02	DONEPEZIL HYDROCHLORIDE	Yes
N07AA02	PYRIDOSTIGMINE BROMIDE	Yes
N07AB02	BETHANECHOL CHLORIDE	Yes
N07BC02	METHADONE HYDROCHLORIDE	Yes
N07CA02	CINNARIZINE	Yes
N07XX02	RILUZOLE	Yes
P01AB02	TINIDAZOLE	Yes
P01BA02	HYDROXYCHLOROQUINE SULFATE	Yes
R03AC02	SALBUTAMOL (AS SULFATE)	Yes
R03AL02	IPRATROPIUM / SALBUTAMOL (AS SULFATE)	Yes
R03BA02	BUDESONIDE	Yes
R03CA02	EPHEDRINE HYDROCHLORIDE	Yes
R03CC02	SALBUTAMOL (AS SULFATE)	Yes
R05CB02	BROMHEXINE HYDROCHLORIDE	Yes
R06AA02	DIPHENHYDRAMINE HYDROCHLORIDE	Yes
R06AD02	PROMETHAZINE	Yes
R06AX02	CYPROHEPTADINE HYDROCHLORIDE	Yes

R07AA02	PORACTANT ALFA	Yes
R07AA02	BERACTANT	Yes
S01ED02	BETAXOLOL	Yes
V03AE02	SEVELAMER HYDROCHLORIDE	Yes
A03CA02	CLIDINIUM / CHLORDIAZEPOXIDE	Yes
A04AA02	GRANISETRON	Yes
C01CA02	ISOPROTERENOL HYDROCHLORIDE	Yes
C07AB02	METOPROLOL SUCCINATE	Yes
C09BA02	ASA / ATORVASTATIN (AS CALCIUM) / ENALAPRIL MALEATE / HYDROCHLOROTHIAZIDE	Yes
G02AD02	PROSTAGLANDIN E2	Yes
G03GB02	CLOMIPHENE	Yes
H03BB02	METHIMAZOLE	Yes
J01MA02	CIPROFLOXACIN (AS LACTATE)	Yes
J04AK02	ETHAMBUTOL HYDROCHLORIDE	Yes
L01EA02	DASATINIB	Yes
L02AE02	LEUPRORELIN ACETATE	No
N04BA02	LEVODOPA / CARBIDOPA	Yes
N06BA02	DEXTROAMPHETAMINE SULFATE	No
R05CB02	BROMHEXINE HYDROCHLORIDE	Yes
R05FA02	EXPECTORANT CODEINE	Yes
R06AA02	DIPHENHYDRAMINE / AMMONIUM CHLORIDE	Yes
R06AD02	PROMETHAZINE HYDROCHLORIDE	Yes
R07AA02	BOVACTANT	No
V03AE02	SEVELAMER CARBONATE	Yes
A02BA03	FAMOTIDINE	Yes
A02BC03	LANSOPRAZOLE	Yes
A03FA03	DOMPERIDONE	Yes
A07DA03	LOPERAMIDE HYDROCHLORIDE	Yes
A10BG03	PIOGLITAZONE	Yes
A10BK03	EMPAGLIFLOZIN	Yes
A11HA03	VITAMIN E	Yes

A12AA03	CALCIUM GLUCONATE	Yes
A16AX03	SODIUM PHENYLBUTYRATE	Yes
B01AA03	Warfarin	Yes
B03BA03	HYDROXOCOBALAMIN	Yes
C01BA03	DISOPYRAMIDE	Yes
C01BC03	PROPAFENONE HYDROCHLORIDE	Yes
C01CA03	NOREPINEPHRINE (AS BITARTRATE)	Yes
C03AA03	HYDROCHLOROTHIAZIDE	Yes
C04AD03	PENTOXIFYLLINE	Yes
C07AB03	ATENOLOL	Yes
C09AA03	LISINOPRIL	Yes
C09CA03	VALSARTAN	Yes
C09DA03	VALSARTAN / HYDROCHLOROTHIAZIDE	Yes
C10BX03	AMLODIPINE (AS BESILATE) / ATORVASTATIN (AS CALCIUM)	Yes
G02CB03	CABERGOLINE	Yes
G03AB03	CONTRACEPTIVE TRIPHASIC	Yes
G03BA03	TESTOSTERONE ENANTHATE	Yes
G03CA03	ESTRADIOL VALERATE	Yes
G03DA03	HYDROXYPROGESTERONE CAPROATE	Yes
G03DC03	LYNESTRENOL	Yes
G04BE03	SILDENAFIL (AS CITRATE)	Yes
G04CA03	TERAZOSIN	Yes
H01BB03	CARBETOCIN	No
H02AA03	DESOXYCORTICOSTERONE ACETATE	Yes
H05BA03	CALCITONIN	Yes
J01GB03	GENTAMICIN	Yes
J02AC03	VORICONAZOLE	Yes
J05AR03	EMTRICITABINE / TENOFOVIR ALAFENAMIDE (AS FUMARATE)	Yes
L01EA03	NILOTINIB	Yes
L02AE03	GOSERELIN ACETATE	Yes
L02BA03	FULVESTRANT	Yes

L02BB03	BICALUTAMIDE	Yes
L02BX03	ABIRATERONE	Yes
L03AB03	INTERFERON GAMMA-1B	Yes
L04AC03	ANAKINRA	Yes
L04AX03	METHOTREXATE SODIUM	Yes
M01AB03	TOLMETIN	Yes
M03BA03	METHOCARBAMOL	Yes
M04AA03	FEBUXOSTAT	Yes
M05BA03	PAMIDRONATE DISODIUM	Yes
N02AB03	FENTANYL (AS CITRATE)	Yes
N02CC03	ZOLMITRIPTAN	Yes
N03AA03	PRIMIDONE	Yes
N04BA03	LEVODOPA / CARBIDOPA / ENTACAPONE	Yes
N05AB03	PERPHENAZINE	Yes
N05AH03	OLANZAPINE	Yes
N05CF03	ZALEPLON	Yes
N06AB03	FLUOXETINE (AS HYDROCHLORIDE)	Yes
N06BX03	PIRACETAM	Yes
N06DA03	RIVASTIGMINE (AS HYDROGEN TARTRATE)	Yes
N07BA03	VARENICLINE TARTRATE	Yes
N07BB03	ACAMPROSATE	No
N07BC03	BUPRENORPHINE (AS HYDROCHLORIDE)	No
P02CA03	ALBENDAZOLE	Yes
R01AX03	IPRATROPIUM BROMIDE	Yes
R03AL03	UMECLIDINIUM (AS BROMIDE) / VILANTEROL (AS TRIFENATATE)	Yes
R03DC03	MONTELUKAST	Yes
R05CA03	GUAIFENESIN	Yes
V03AF03	CALCIUM FOLINATE	Yes
A01AC03	HYDROCORTISONE	Yes
A11AA03	MULTIVITAMIN THERAPEUTIC	Yes
C09BA03	LISINOPRIL / HYDROCHLOROTHIAZIDE	Yes

J01GB03	GENTAMICIN	Yes
J05AG03	EFAVIRENZ	Yes
L01EX03	PAZOPANIB (AS HYDROCHLORIDE)	Yes
L03AA03	POTASSIUM CITRATE	Yes
M05BB03	ALENDRONIC ACID / COLECALCIFEROL	Yes
N06AB03	FLUOXETINE (AS HYDROCHLORIDE)	No
R01AC03	AZELASTINE HYDROCHLORIDE	Yes
S01EB03	ECHOTHIOPHATE IODIDE	No
A02AA04	MAGNESIUM HYDROXIDE	Yes
A02BC04	RABEPRAZOLE SODIUM	Yes
A03AA04	MEBEVERINE HYDROCHLORIDE	Yes
A10AB04	INSULIN LISPRO	Yes
A10AE04	INSULIN GLARGINE	Yes
A11CC04	CALCITRIOL	Yes
A12AA04	CALCIUM CARBONATE	Yes
A16AX04	NITISINONE	Yes
B01AB04	DALTEPARIN SODIUM	Yes
B01AC04	CLOPIDOGREL (AS BISULFATE)	Yes
B02BX04	ROMIPLOSTIM	Yes
C01BC04	FLECAINIDE ACETATE	Yes
C01CA04	DOPAMINE HYDROCHLORIDE	Yes
C02KX04	MACITENTAN	Yes
C03DA04	EPLERENONE	Yes
C09DX04	SACUBITRIL/VALSARTAN	Yes
C10AB04	GEMFIBROZIL	Yes
G01AF04	MICONAZOLE NITRATE	Yes
G02AD04	CARBOPROST	Yes
G03DA04	PROGESTERONE	Yes
G03FA04	CONTRACEPTIVE HD	Yes
G03GA04	UROFOLLITROPIN	Yes
G04BD04	OXYBUTYNIN HYDROCHLORIDE	Yes

G04BE04	YOHIMBINE HYDROCHLORIDE	Yes
H02AB04	METHYLPREDNISOLONE ACETATE	Yes
J01CA04	AMOXICILLIN	Yes
J01DB04	CEFAZOLIN	Yes
J01DD04	CEFTRIAXONE (AS SODIUM)	Yes
J01XX04	SPECTINOMYCIN	Yes
J02AC04	POSACONAZOLE	Yes
J02AX04	CASPOFUNGIN ACETATE	Yes
L02AE04	TRIPTORELIN	Yes
L02BG04	LETROZOLE	Yes
L04AA04	ANTITHYMOCYTE IMMUNOGLOBULIN	Yes
L04AB04	INFLIXIMAB	Yes
L04AX04	LENALIDOMIDE	Yes
M05BA04	ALENDRONATE	Yes
M05BX04	DENOSUMAB	Yes
N02AB04	FENTANYL	Yes
N02CC04	RIZATRIPTAN	Yes
N03AG04	VIGABATRIN	Yes
N04BC04	ROPINIROLE (AS HYDROCHLORIDE)	Yes
N05AF04	THIOTHIXENE	Yes
N05AH04	QUETIAPINE FUMARATE	Yes
N05BA04	OXAZEPAM	Yes
N06AA04	CLOMIPRAMINE HYDROCHLORIDE	Yes
N06AB04	CITALOPRAM	Yes
N06AF04	TRANYLCYPROMINE	Yes
N06BA04	METHYLPHENIDATE HYDROCHLORIDE	Yes
N06DA04	GALANTAMINE (AS HYDROBROMIDE)	Yes
N07BB04	NALTREXONE HYDROCHLORIDE	Yes
R01AA04	PHENYLEPHRINE HYDROCHLORIDE	Yes
R03BB04	TIOTROPIUM BROMIDE	Yes
R03DA04	THEOPHYLLINE	Yes

R06AA04	CLEMASTINE	Yes
R06AB04	CHLORPHENIRAMINE MALEATE	Yes
S01GA04	OXYMETAZOLINE HYDROCHLORIDE	No
A11AA04	TRACE METAL	Yes
A16AA04	CYSTEAMINE	Yes
C09DX04	SACUBITRIL / VALSARTAN	Yes
L01EB04	osimertinib mesylate	Yes
L01EX04	vandetanib	Yes
M05BA04	ALENDRONATE	Yes
N02CC04	RIZATRIPTAN (AS BENZOATE)	Yes
N06DA04	GALANTAMINE	Yes
S01EC04	BRINZOLAMIDE	Yes
A02BC05	ESOMEPRAZOLE	Yes
A02BX05	BISMUTH SUBCITRATE	Yes
A10AE05	INSULIN DETEMIR	Yes
A10BH05	LINAGLIPTIN	Yes
A11CC05	VITAMIN D3	Yes
A14AA05	OXYMETHOLONE	Yes
A16AA05	CARGLUMIC ACID	Yes
A16AB05	LARONIDASE	Yes
B01AB05	ENOXAPARIN SODIUM	Yes
B01AC05	TICLOPIDINE HYDROCHLORIDE	Yes
C01AA05	DIGOXIN	Yes
C07AA05	PROPRANOLOL HYDROCHLORIDE	Yes
C08CA05	NIFEDIPINE	Yes
C10AA05	ATORVASTATIN (AS CALCIUM)	Yes
C10AB05	FENOFIBRATE	Yes
G03GA05	FOLLITROPIN ALFA	Yes
J01CR05	PIPERACILLIN (AS SODIUM) / TAZOBACTAM (AS SODIUM)	Yes
J05AF05	LAMIVUDINE	Yes
L03AB05	INTERFERON ALFA-2B	Yes

L04AX05	PIRFENIDONE	Yes
M01AB05	DICLOFENAC SODIUM	Yes
N02AA05	OXYCODONE HYDROCHLORIDE	Yes
N04BC05	PRAMIPEXOLE DIHYDROCHLORIDE	Yes
N06AB05	CITALOPRAM	Yes
N06AX05	TRAZODONE HYDROCHLORIDE	Yes
R01AA05	OXYMETAZOLINE HYDROCHLORIDE	Yes
R03BA05	FLUTICASONE PROPIONATE	Yes
R03DA05	AMINOPHYLLINE	Yes
R03DX05	OMALIZUMAB	Yes
A02BX05	BISMUTH SUBCITRATE EQU. TO BISMUTH OXIDE	Yes
A10AB05	INSULIN ASPART	Yes
B02BX05	ELTROMBOPAG (AS OLAMINE)	Yes
C10BA05	ATORVASTATIN (AS CALCIUM) / EZETIMIBE	Yes
L01EX05	REGORAFENIB	Yes
M01AB05	DICLOFENAC SODIUM	Yes
N04BC05	PRAMIPEXOLE DIHYDROCHLORIDE	Yes
N06AB05	PAROXETINE (AS HYDROCHLORIDE)	Yes
R01AD05	BUDESONIDE	Yes
V03AG05	PHOSPHATE SODIUM	Yes
A16AA06	BETAINE	Yes
A16AX06	MIGLUSTAT	Yes
B01AC06	ASA (ACETYLSALICYLIC ACID)	Yes
B05XA06	PHOSPHATE, POTASSIUM MONOBASIC	Yes
C01CA06	PHENYLEPHRINE HYDROCHLORIDE	Yes
C08CA06	NIMODIPINE	Yes
G03AC06	MEDROXYPROGESTERONE ACETATE	Yes
G03GA06	FOLLITROPIN BETA	Yes
G04BX06	PHENAZOPYRIDINE HYDROCHLORIDE	Yes
H02AB06	PREDNISOLONE	Yes
J01GB06	AMIKACIN (AS SULFATE)	Yes

J05AB06	GANCICLOVIR	Yes
L02BG06	Exemestan	Yes
L04AA06	MYCOPHENOLATE MOFETIL	Yes
M01AC06	MELOXICAM	Yes
N02AJ06	ACETAMINOPHEN / CODEINE PHOSPHATE	Yes
N05AB06	TRIFLUOPERAZINE	Yes
N05BA06	LORAZEPAM	Yes
N06AA06	TRIMIPRAMINE (AS MALEATE)	Yes
N06AB06	SERTRALINE (AS HYDROCHLORIDE)	Yes
N07XX06	TETRABENAZINE	Yes
R03AK06	SALMETEROL (AS XINAFOATE) / FLUTICASONE PROPIONATE	Yes
A07AA06	PAROMOMYCIN	Yes
A07EA06	BUDESONIDE	Yes
G02AD06	MISOPROSTOL	Yes
N06AA06	Trimipramine	No
J01CF06	NAFCILLIN	No
A03AA07	DICYCLOMINE HYDROCHLORIDE	Yes
A10BD07	SITAGLIPTIN (AS PHOSPHATE) / METFORMIN HYDROCHLORIDE	Yes
A16AB07	ALGLUCOSIDASE ALFA	Yes
B01AB07	DALTEPARIN	Yes
B01AC07	DIPYRIDAMOLE	Yes
B01AD07	RETEPLASE	Yes
B01AE07	DABIGATRAN ETEXILATE (AS MESYLATE)	Yes
C01CA07	DOBUTAMINE	Yes
C07AA07	SOTALOL HYDROCHLORIDE	Yes
C07AB07	BISOPROLOL FUMARATE	Yes
C10AA07	ROSUVASTATIN (AS CALCIUM)	Yes
G04BD07	TOLTERODINE TARTRATE	Yes
J01AA07	TETRACYCLINE HYDROCHLORIDE	Yes
J01DD07	CEFTIZOXIME	Yes
J05AF07	TENOFOVIR	Yes

L03AB07	INTERFERON BETA-1A	Yes
L04AC07	TOCILIZUMAB	Yes
L04AX07	DIMETHYL FUMARATE	Yes
N06BA07	MODAFINIL	Yes
N07XX07	FAMPRIDINE	Yes
R03AK07	BUDESONIDE / FORMOTEROL FUMARATE	Yes
R06AE07	CETIRIZINE HYDROCHLORIDE	Yes
V03AF07	RASBURICASE	Yes
C01CA07	DOBUTAMINE HCL	Yes
C07BB07	BISOPROLOL FUMARATE / HYDROCHLOROTHIAZIDE	Yes
C07FB07	AMLODIPINE (AS BESILATE) / BISOPROLOL FUMARATE	Yes
C09CA07	TELMISARTAN	Yes
G03GA07	LUTROPIN ALFA	Yes
J05AP07	DACLATASVIR	Yes
M09AX07	NUSINERSEN (AS SODIUM)	No
N04BC07	APOMORPHINE HYDROCHLORIDE	Yes
R03BA07	MOMETASONE FUROATE	Yes
V03AE07	CALCIUM ACETATE	No
A10AB08	INSULIN GLULISINE	Yes
C01DA08	ISOSORBIDE DINITRATE	Yes
G03DB08	DIENOGEST	Yes
G03GA08	CHORIOGONADOTROPIN ALFA	Yes
G04BD08	SOLIFENACIN SUCCINATE	Yes
G04BE08	TADALAFIL	Yes
H02AB08	TRIAMCINOLONE ACETONIDE	Yes
J01CE08	PENICILLIN G BENZATHIN	Yes
J01DD08	CEFIXIME	Yes
J01XX08	LINEZOLID	Yes
J05AF08	ADEFOVIR DIPIVOXIL	Yes
L03AB08	INTERFERON BETA-1B	Yes
M05BA08	ZOLEDRONIC ACID	Yes

N05AX08	RISPERIDONE	Yes
N05CD08	MIDAZOLAM	Yes
N06AB08	FLUVOXAMINE MALEATE	Yes
R01AA08	NAPHAZOLINE HYDROCHLORIDE	Yes
R01AD08	FLUTICASONE PROPIONATE	Yes
R03AK08	BECLOMETHASONE DIPROPIONATE / FORMOTEROL FUMARATE	Yes
G04BE08	TADALAFIL	Yes
J05AP08	SOFOSBUVIR	Yes
A10BB09	GLICLAZIDE	Yes
C07AB09	ESMOLOL HYDROCHLORIDE	No
C10AX09	EZETIMIBE	Yes
G03AA09	CONTRACEPTIVE DE	Yes
G03GA09	CORIFOLLITROPIN ALFA	Yes
H02AB09	HYDROCORTISONE (AS SODIUM SUCCINATE)	Yes
J01CE09	PENICILLIN G PROCAINE	Yes
J01FA09	CLARITHROMYCIN	Yes
N03AX09	LAMOTRIGINE	Yes
N05BA09	CLOBAZAM	Yes
N06AA09	AMITRIPTYLINE HYDROCHLORIDE	Yes
N06BA09	ATOMOXETINE HYDROCHLORIDE	Yes
R01AD09	MOMETASONE FUROATE	Yes
R05DA09	DEXTROMETHORPHAN HYDROBROMIDE	Yes
R03AK09	MOMETASONE / FORMOTEROL	Yes
R06AE09	LEVOCETIRIZINE DIHYDROCHLORIDE	Yes
J05AF10	ENTECAVIR	No
L03AB10	PEGINTERFERON ALFA-2B	Yes
C01EB10	ADENOSINE	Yes
D11AX10	FINASTERIDE	Yes
G01AA10	CLINDAMYCIN (AS PHOSPHATE)	Yes
J01CE10	PENICILLIN V BENZATHINE	Yes
J01FA10	AZITHROMYCIN	Yes

J05AF10	ENTECAVIR	Yes
J05AR10	LOPINAVIR / RITONAVIR	Yes
L04AA10	SIROLIMUS	Yes
N06AA10	NORTRIPTYLINE	Yes
N06AB10	ESCITALOPRAM (AS OXALATE)	Yes
R05CA10	EXPECTORANT	Yes
A06AD11	LACTULOSE	Yes
B01AD11	TENECTEPLASE	No
A07AA11	RIFAXIMIN	Yes
A10BD11	METFORMIN HYDROCHLORIDE / LINAGLIPTIN	Yes
A16AX11	Sodium Benzoate	No
C03BA11	INDAPAMIDE	Yes
G01AX11	POVIDONE IODINE	Yes
J05AB11	VALACYCLOVIR	Yes
L03AB11	PEGINTERFERON ALFA-2A	Yes
N03AX11	TOPIRAMATE	Yes
N06AX11	MIRTAZAPINE	Yes
R06AA11	DIMENHYDRINATE	Yes
A04AD12	APREPITANT	Yes
A16AB12	ELOSULFASE ALFA	Yes
G03FA12	MEDROXYPROGESTERONE ACETATE / ESTRADIOL CYPIONATE	Yes
J01AA12	TIGECYCLINE	Yes
J01CA12	PIPERACILLIN (AS SODIUM) / TAZOBACTAM (AS SODIUM)	Yes
J01MA12	LEVOFLOXACIN	Yes
N03AX12	GABAPENTIN	Yes
N05AX12	ARIPIPRAZOLE	Yes
N05BA12	ALPRAZOLAM	Yes
N06AA12	DOXEPIN	Yes
N06AX12	BUPROPION HYDROCHLORIDE	Yes
R03AC12	SALMETEROL (AS XINAFOATE)	Yes
A16AX12	TRIENTINE HYDROCHLORIDE	Yes

N06BA12	LISDEXAMFETAMINE MESILATE	Yes
C10AX13	EVOLOCUMAB	Yes
G03FA13	MEDROXYPROGESTERONE ACETATE / ESTRADIOL CYPIONATE	Yes
L03AA13	PEGFILGRASTIM	Yes
L03AX13	GLATIRAMER ACETATE	Yes
L04AA13	LEFLUNOMIDE	Yes
R03AC13	FORMOTEROL FUMARATE	Yes
R06AX13	LORATADINE	Yes
A03AX13	SIMETHICONE (DIMETHICONE ACTIVATED)	Yes
J05AF13	TENOFOVIR ALAFENAMIDE (AS FUMARATE)	Yes
G04BX14	DAPOXETINE (AS HYDROCHLORIDE)	Yes
J01MA14	MOXIFLOXACIN (AS HYDROCHLORIDE)	Yes
J05AB14	VALGANCICLOVIR	Yes
N03AX14	LEVETIRACETAM	Yes
N07XX14	EDARAVONE	Yes
A06AD15	POLYETHYLENE GLYCOL	Yes
J01MA15	GEMIFLOXACIN	Yes
M01AB15	KETOROLAC TROMETAMOL	Yes
N03AX15	ZONISAMIDE	Yes
A06AD15	POLYETHYLENE GLYCOL - ELECTROLYTE 1	Yes
A10BD15	DAPAGLIFLOZIN (AS PROPANEDIOL) / METFORMIN HYDROCHLORIDE	Yes
B01AC16	EPTIFIBATIDE	Yes
C01DX16	NICORANDIL	Yes
J05AB16	REMDESIVIR	Yes
L03AX16	PLERIXAFOR	Yes
N03AX16	PREGABALIN	Yes
N06AX16	VENLAFAXINE	Yes
B01AC17	TIROFIBAN	No
C01CA17	MIDODRINE HYDROCHLORIDE	Yes
C01EB17	IVABRADINE (AS HYDROCHLORIDE)	Yes
G03FA17	DROSPIRENONE / ETHINYL ESTRADIOL	Yes

J01CA17	TEMOCILLIN SODIUM	No
N02AJ17	ACETAMINOPHEN / OXYCODONE HYDROCHLORIDE	Yes
R06AX17	KETOTIFEN	Yes
A06AD18	SORBITOL	Yes
C01EB18	RANOLAZINE	Yes
L04AA18	EVEROLIMUS	Yes
N03AX18	LACOSAMIDE	Yes
N05CM18	DEXMEDETOMIDINE (AS HYDROCHLORIDE)	Yes
N05CM18	DEXMEDETOMIDINE HYDROCHLORIDE	Yes
A10BD19	EMPAGLIFLOZIN / LINAGLIPTIN	Yes
G01A	CLINDAMYCIN (AS PHOSPHATE) / CLOTRIMAZOLE	Yes
A10BD20	EMPAGLIFLOZIN / METFORMIN HYDROCHLORIDE	Yes
R05DA20	DEXTROMETHORPHAN / PSEUDOEPHEDRINE	Yes
N06AA21	MAPROTILINE HYDROCHLORIDE	Yes
N06AX21	DULOXETINE	Yes
B01AC22	PRASUGREL	Yes
N06AX22	AGOMELATINE	Yes
L04AA23	NATALIZUMAB	Yes
D11AX23	POTASSIUM AMINOBENZOIC ACID (POTABA)	Yes
J05AR23	ATAZANAVIR / RITONAVIR	Yes
B01AC24	TICAGRELOR	Yes
C01CA24	EPINEPHRINE	Yes
R06AX26	FEXOFENADINE HYDROCHLORIDE	Yes
C01CA26	EPHEDRINE HYDROCHLORIDE	Yes
J05AX27	FAVIPIRAVIR	Yes
L04AA27	FINGOLIMOD	Yes
R06AX27	DESLORATADINE SYRUP ORAL 2.5 mg/5 mL 120MILLILITER	Yes
A10BD27	EMPAGLIFLOZIN / LINAGLIPTIN / METFORMIN HYDROCHLORIDE	Yes
L04AA29	TOFACITINIB (AS CITRATE)	Yes
G03GA30	MENOTROPINS	Yes
A10AB30	INSULIN BIPHASIC ISOPHANE	Yes

G03GA30	FOLLITROPIN ALFA / LUTROPIN ALFA	Yes
L04AA31	TERIFLUNOMIDE	No
L04AA31	TERIFLUNOMIDE	Yes
L04AA34	ALEMTUZUMAB	Yes
L04AA36	OCRELIZUMAB	Yes
L04AA37	BARICITINIB	Yes
B03A	FERROUS-GLYCINE-SULFATE	No
L04AA40	CLADRIBINE	Yes
N02BA51	ACETAMINOPHEN / CAFFEINE / ASA	Yes
N02CC51	SUMATRIPTAN / NAPROXEN	Yes
J01DH51	IMIPENEM / CILASTATIN	Yes
J05AP51	LEDIPASVIR / SOFOSBUVIR	Yes
N02BA51	ASA / ASCORBIC ACID	Yes
N02BE51	ACETAMINOPHEN / IBUPROFEN	Yes
N07BC51	BUPRENORPHINE / NALOXONE	Yes
S01ED51	DORZOLAMIDE (AS HYDROCHLORIDE) / TIMOLOL (AS MALEATE)	Yes
G04CA52	DUTASTERIDE / TAMSULOSIN	Yes
N03AB52	PHENYTOIN / PHENOBARBITAL	Yes
N01BB52	ANTIHEMORRHOID	Yes
A02BA53	FAMOTIDINE / CALCIUM CARBONATE / MAGNESIUM HYDROXIDE	Yes
R01BA53	ANTIHISTAMINE DECONGESTANT (2)	Yes
G04CA53	SOLIFENACIN SUCCINATE / TAMSULOSIN HYDROCHLORIDE	Yes
R01BA53	GUAIFENESIN / CHLORPHENIRAMINE / PHENYLEPHRINE	Yes
R03DA54	THEOPHYLLINE / GUAIFENESIN	Yes
S01EC54	BRINZOLAMIDE / TIMOLOL (AS MALEATE)	Yes
J05AP55	SOFOSBUVIR / VELPATASVIR	Yes
G03CA57	CONJUGATED ESTROGENS	Yes
R01AD58	AZELASTINE / FLUTICASONE	Yes
J05AP59	DACLATASVIR / SOFOSBUVIR	Yes
N02AA59	CODEINE PHOSPHATE / GUAIFENESIN / PHENYLEPHRINE	Yes
R05X	ADULT COLD PREPARATIONS (4-3)	Yes

N02BE71	ACETAMINOPHEN / CAFFEINE	Yes
N02BE71	ACETAMINOPHEN / CAFFEINE / IBUPROFEN	Yes
N02CA72	ERGOTAMINE TARTRATE / CAFFEINE	Yes
B03AC	IRON (AS SUCROSE)	Yes
A11EA	VITAMIN B COMPLEX	Yes
A11EX	VITAMIN B1 / VITAMIN B6 / VITAMIN B12	No
G03FA	CHLORMADINONE / ETHINYL ESTRADIOL	Yes

Class A had the highest precent of DID in the 1st ATC level.

The highest precent of DID in the 1st ATC level in Total Utilization

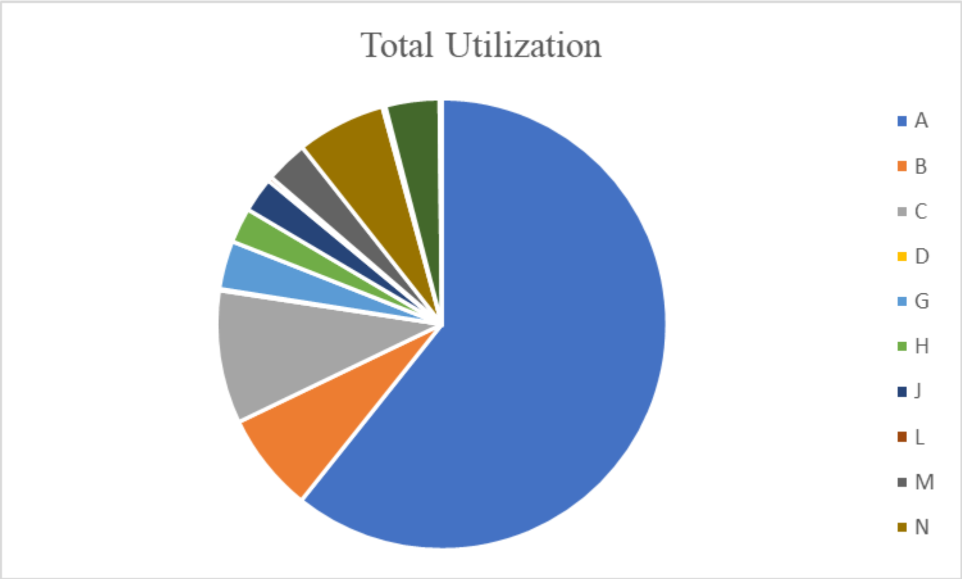

The highest precent of DID in the 1st ATC level in Utilization under Insurance Coverage

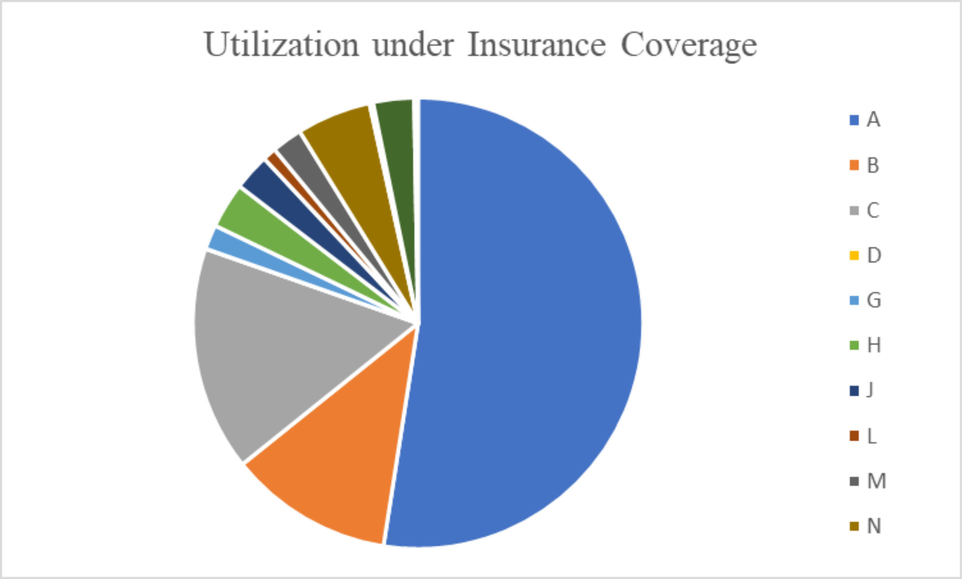

Appendix 2. The highest precent of DID in the 1st ATC level

DID utilization Annual Percentage Change (APC)

Based on the APC index, utilization trends in the 1st ATC level was as following figure:

Alimentary Tract And Metabolism (A)

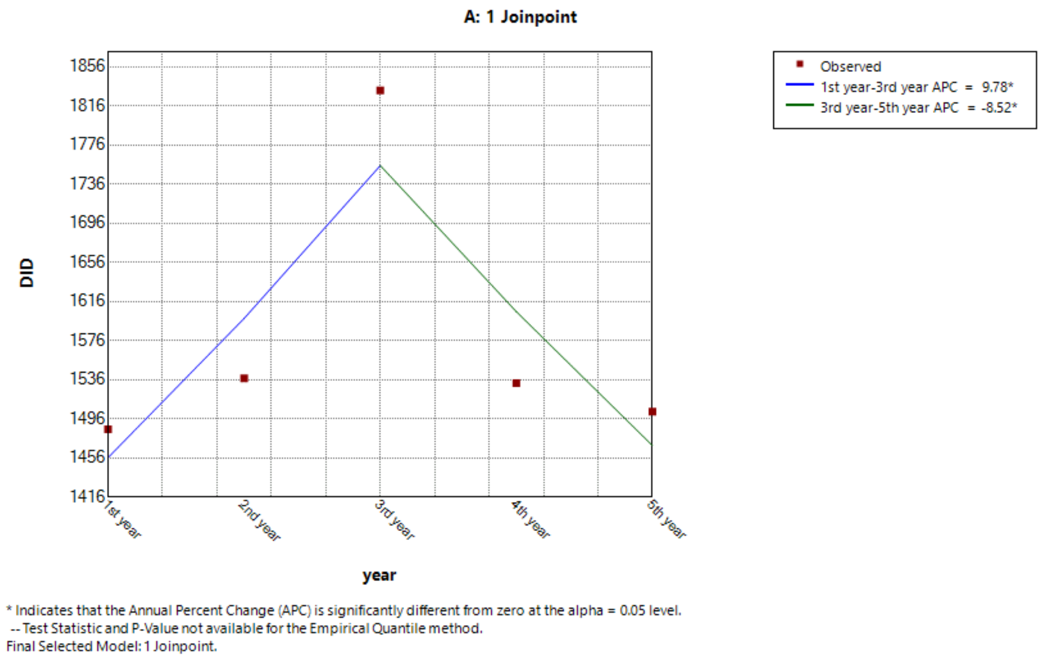

Appendix 3. Trend total utilization of class A

Cardiovascular System (C)

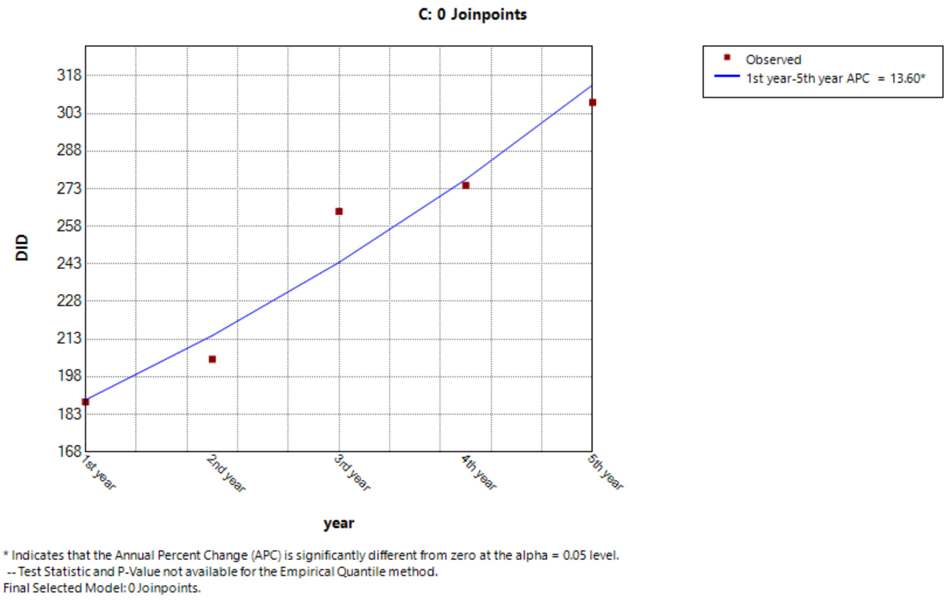

Appendix 4. Trend total utilization of class C

Dermatologicals (D)

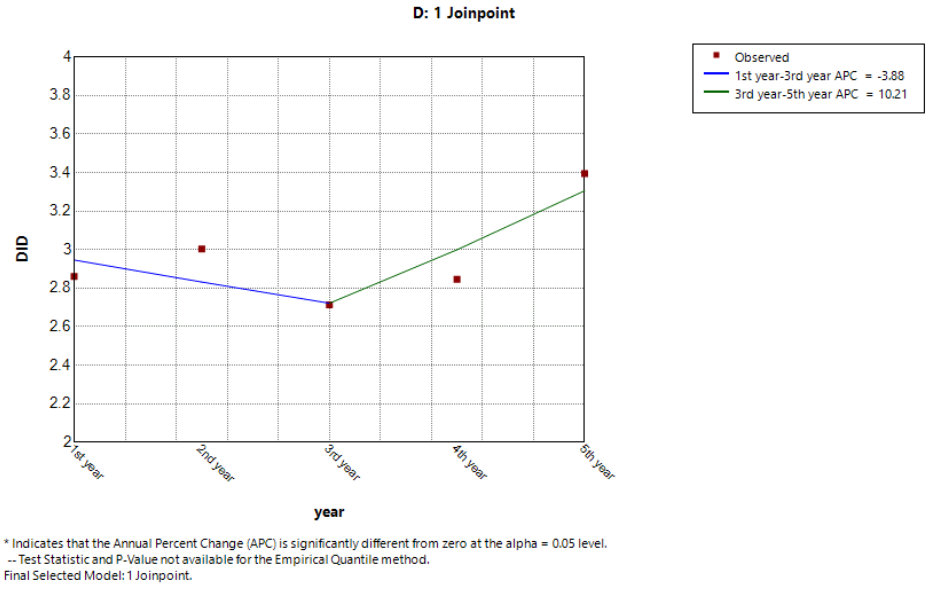

Appendix 5. Trend total utilization of class D

Genito Urinary System and Sex Hormones (G)

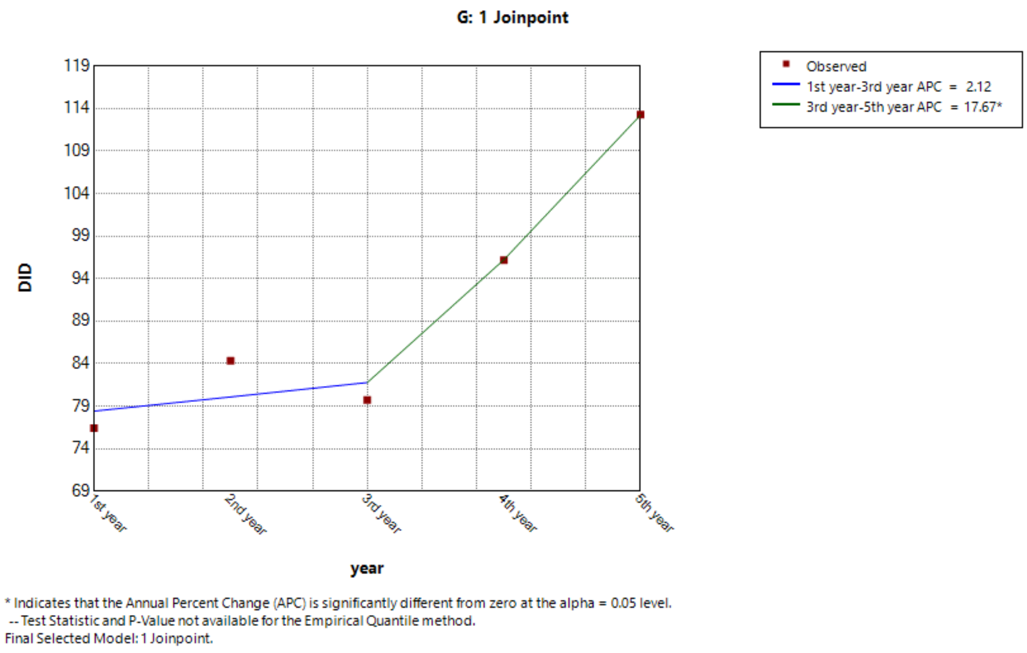

Appendix 6. Trend total utilization of class G

Systemic Hormonal Preparations, Excl. Sex Hormones and Insulins (H)

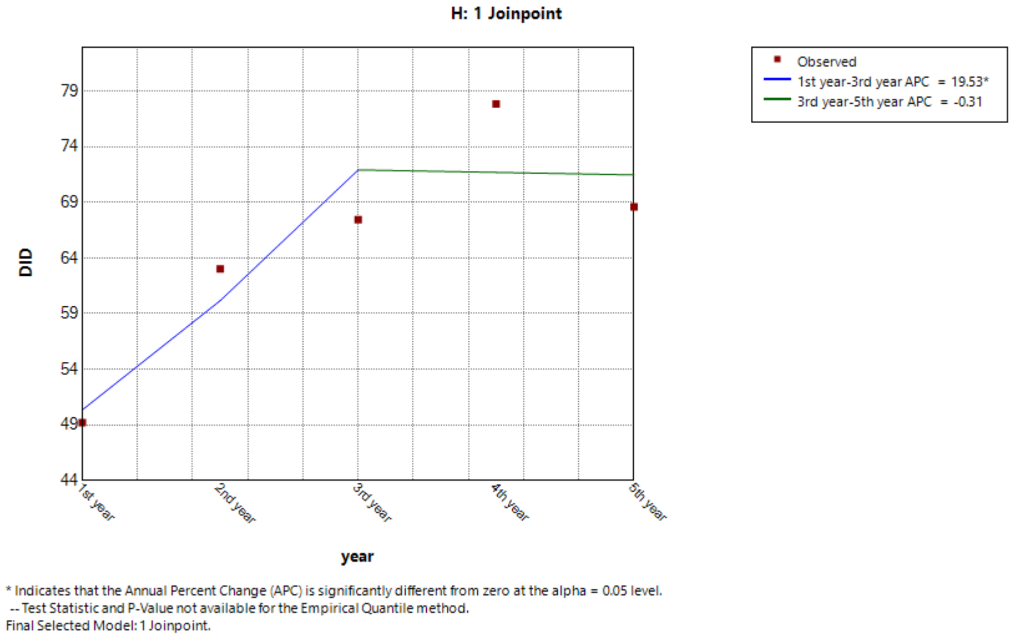

Appendix 7. Trend total utilization of class H

Antiinfectives For Systemic Use (J)

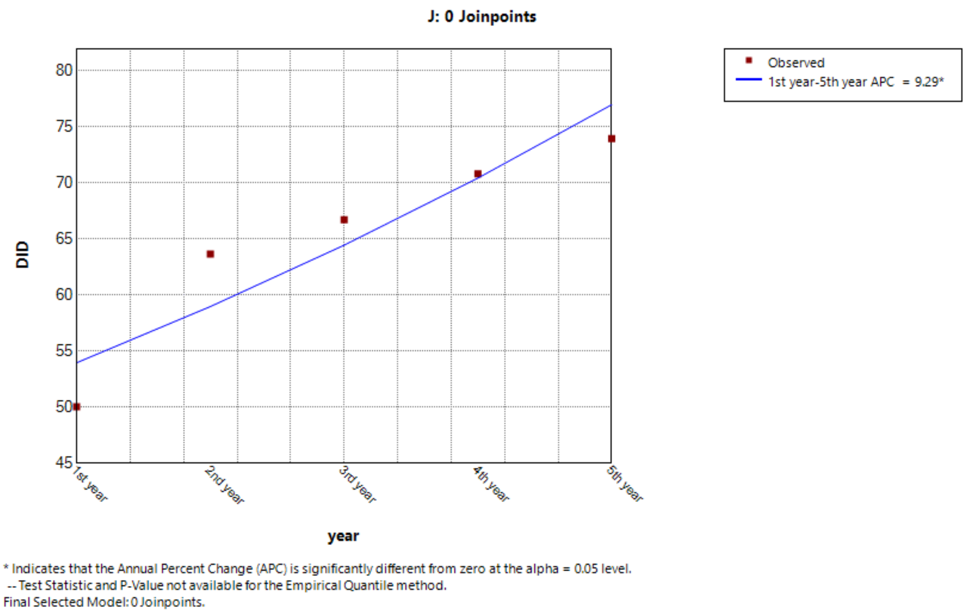

Appendix 8. Trend total utilization of class J

Antineoplastic And Immunomodulating Agents (L)

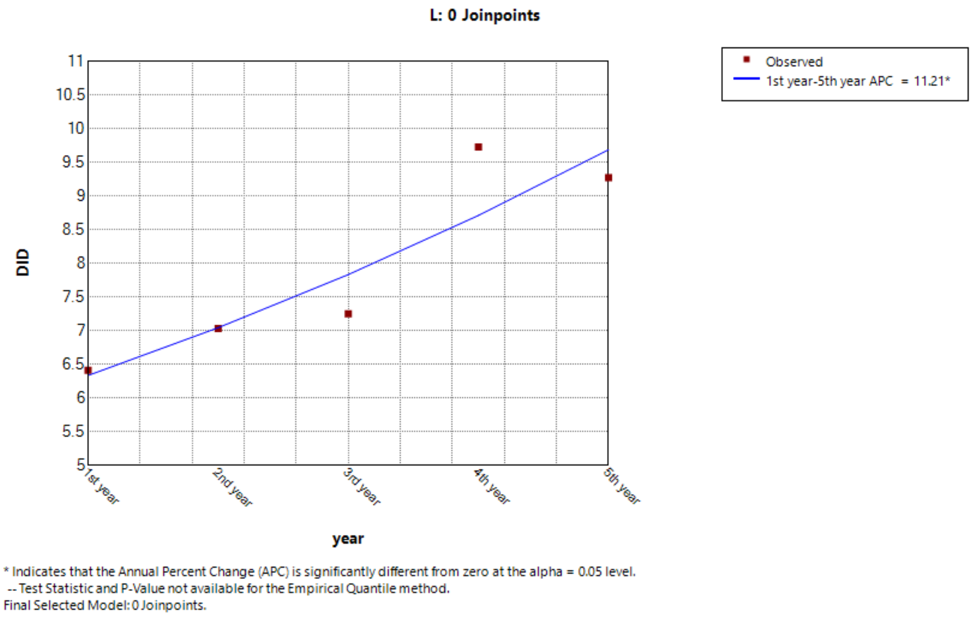

Appendix 9. Trend total utilization of class L

Nervous System (N)

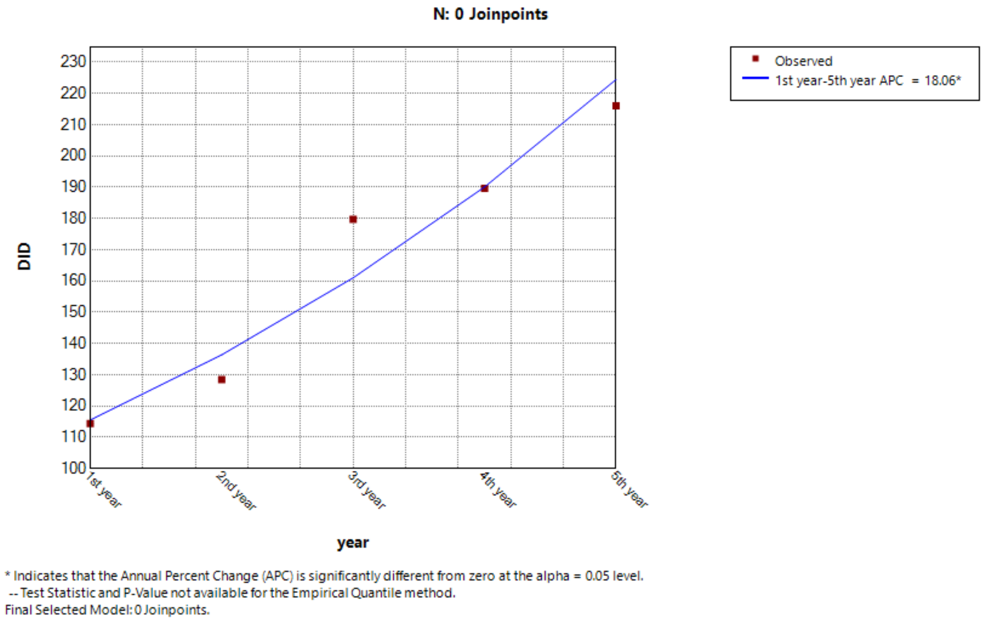

Appendix 10. Trend total utilization of class N

Antiparasitic Products, Insecticides and Repellents (P)

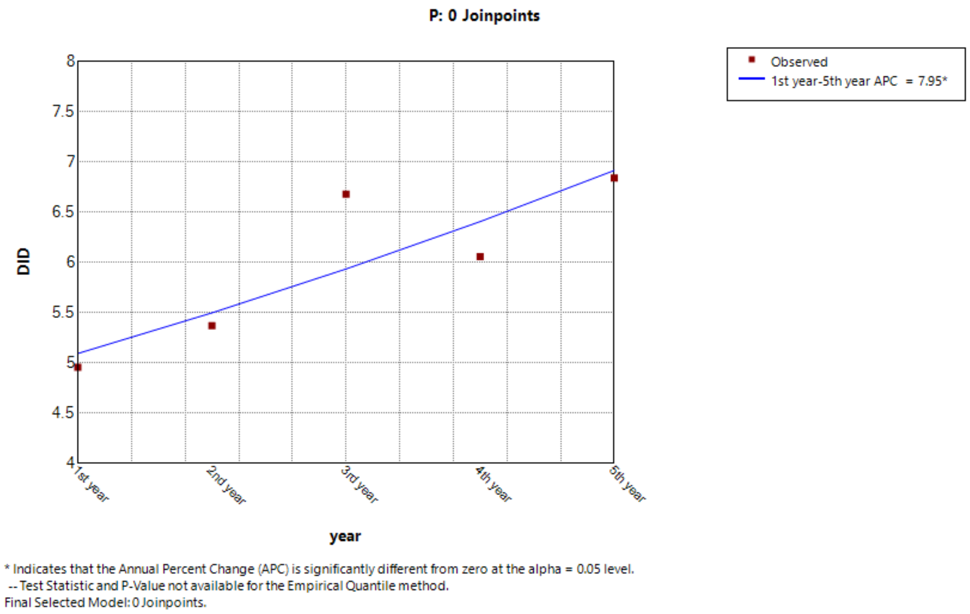

Appendix 11. Trend total utilization of class P

Sensory Organs (S)

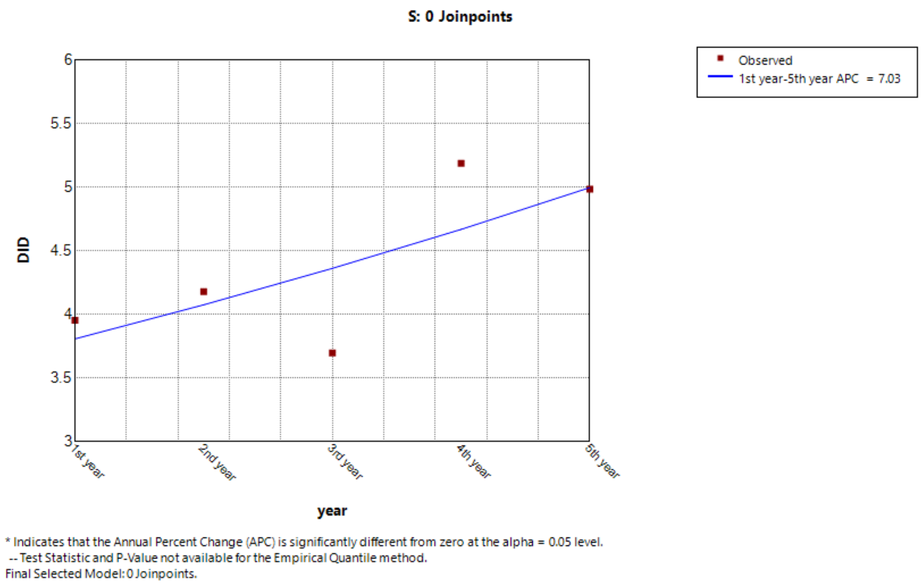

Appendix 12. Trend total utilization of class S

Various (V)

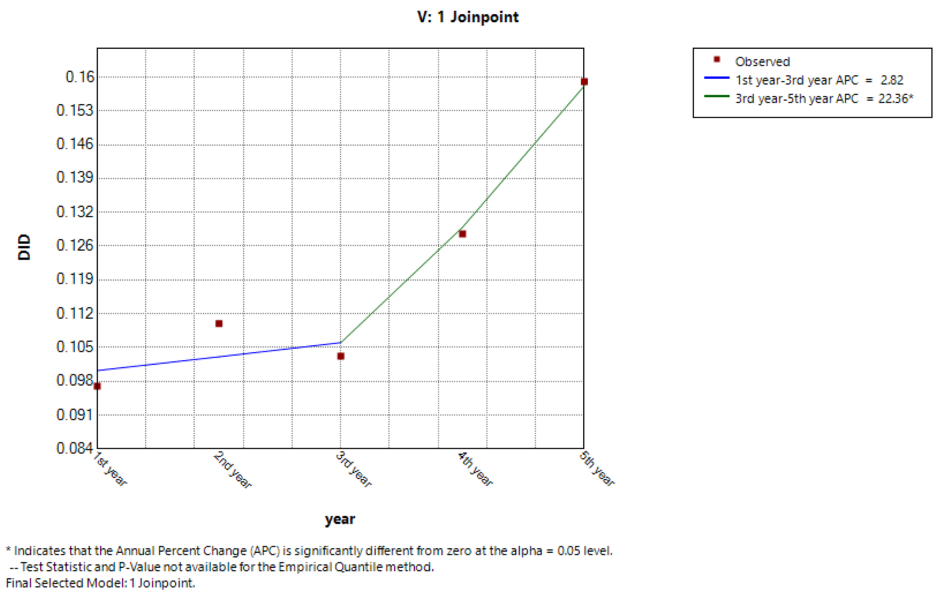

Appendix 13. Trend total utilization of class V

Joinpoint regression Analysis of pharmaceutical DID utilization under insurance coverage from march 2018 to march 2023

Alimentary Tract and Metabolism (A)

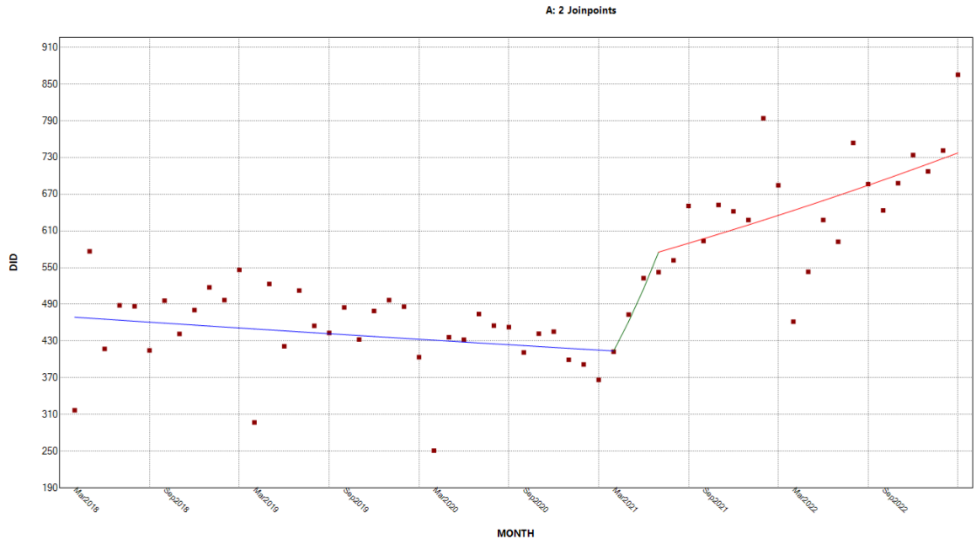

Appendix 14. Trend of utilization under insurance coverage in class A

Blood and blood forming organs (B)

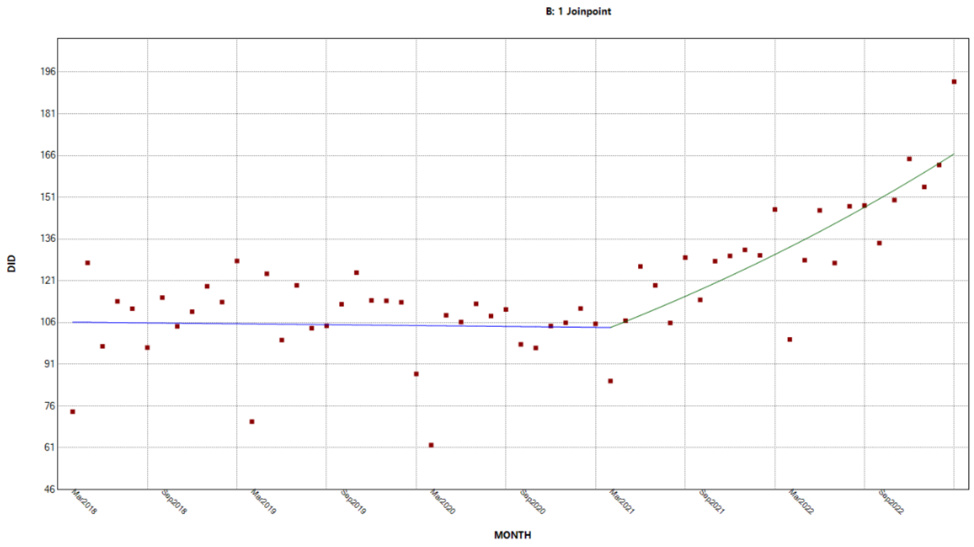

Appendix 15. Trend of utilization under insurance coverage in class B

Cardiovascular System (C)

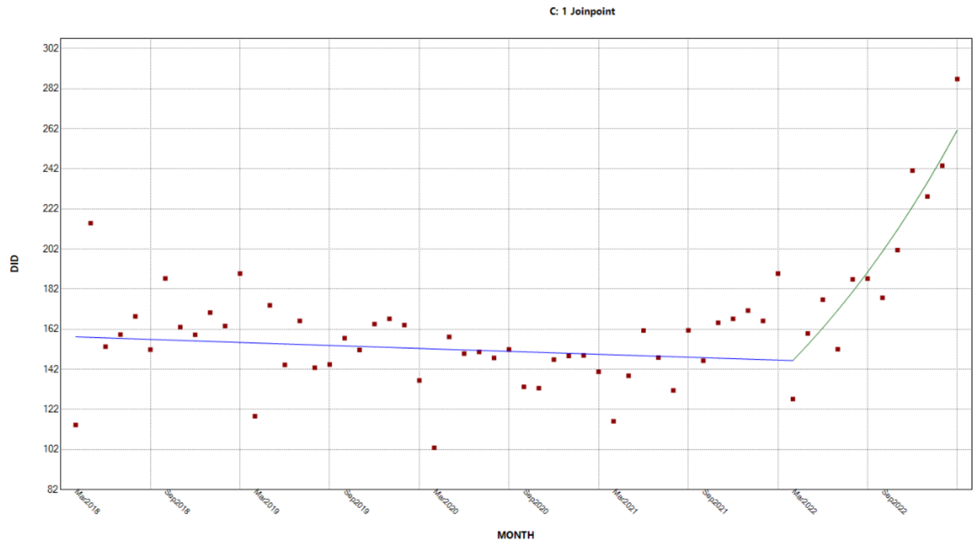

Appendix 16. Trend of utilization under insurance coverage in class C

Dermatologicals (D)

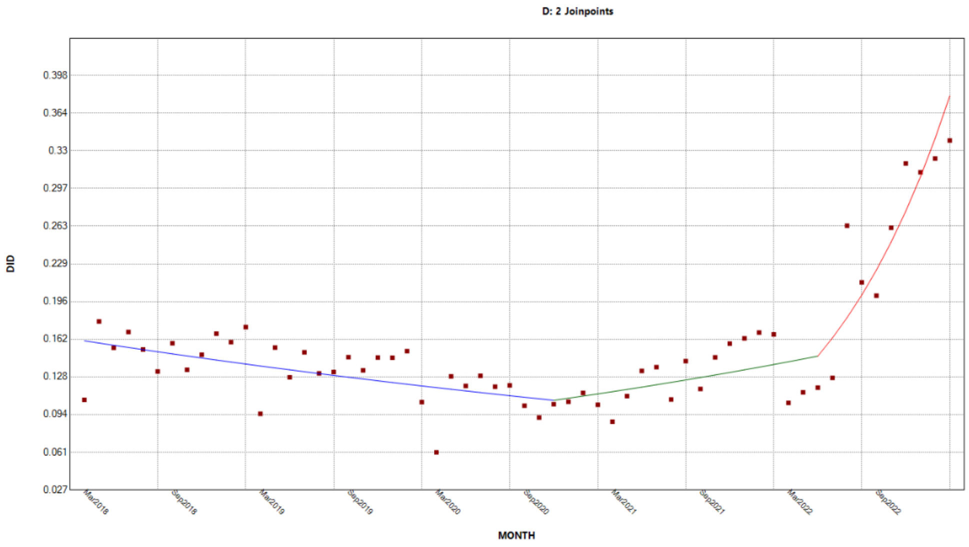

Appendix 17. Trend of utilization under insurance coverage in class D

Genito Urinary System and Sex Hormones (G)

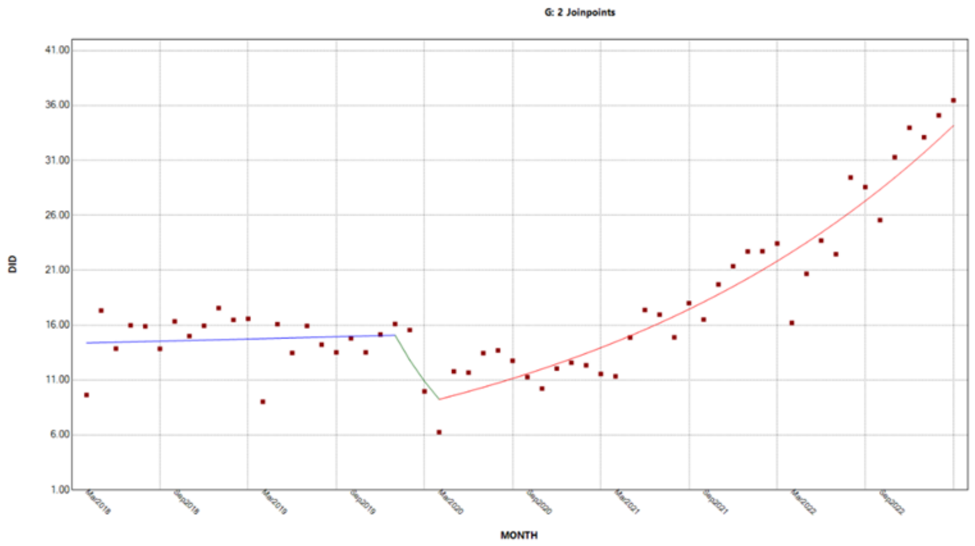

Appendix 18. Trend of utilization under insurance coverage in class G

Systemic Hormonal Preparations, Excl. Sex Hormones and Insulins (H)

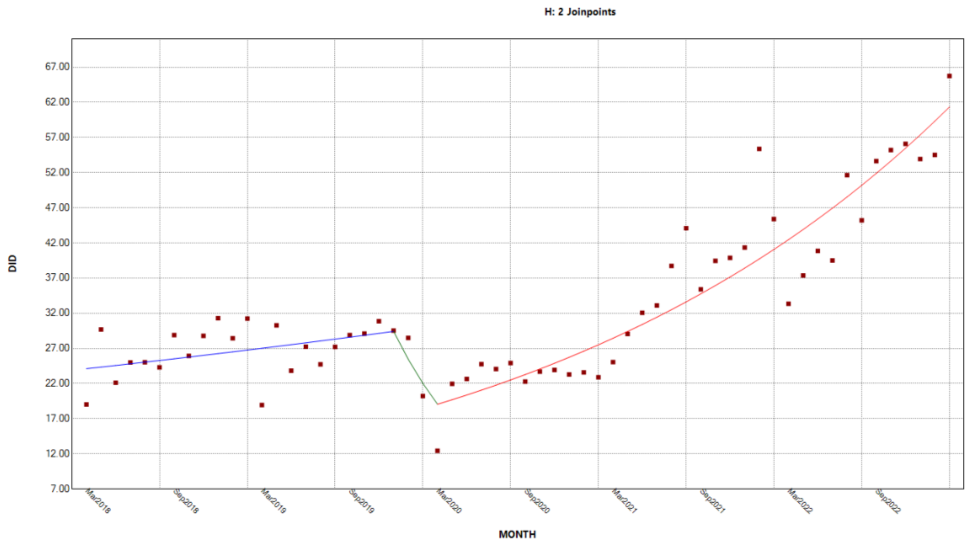

Appendix 19. Trend of utilization under insurance coverage in class H

Antineoplastic and Immunomodulating Agents (L)

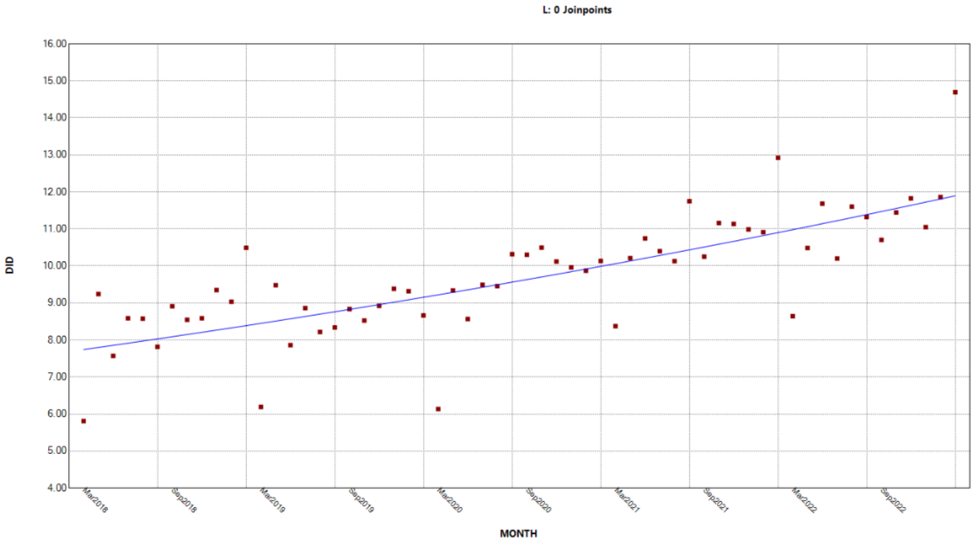

Appendix 20. Trend of utilization under insurance coverage in class L

Musculo-Skeletal System (M)

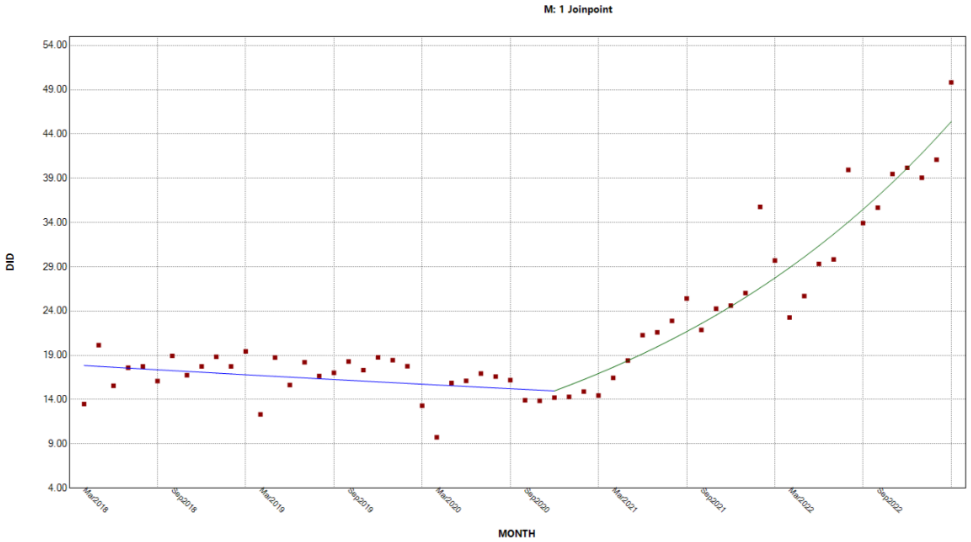

Appendix 21. Trend of utilization under insurance coverage in class M

Nervous System (N)

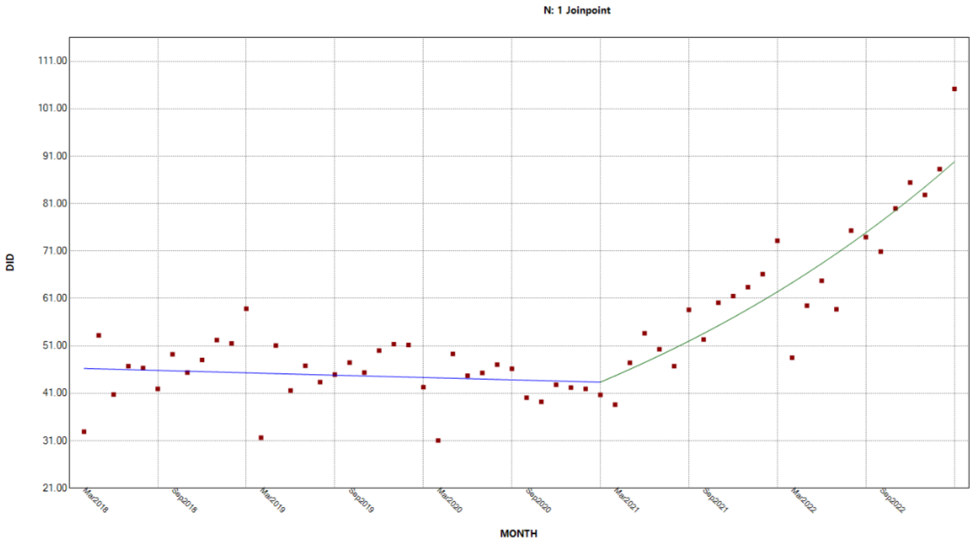

Appendix 22. Trend of utilization under insurance coverage in class N

Antiparasitic Products, Insecticides and Repellents (P)

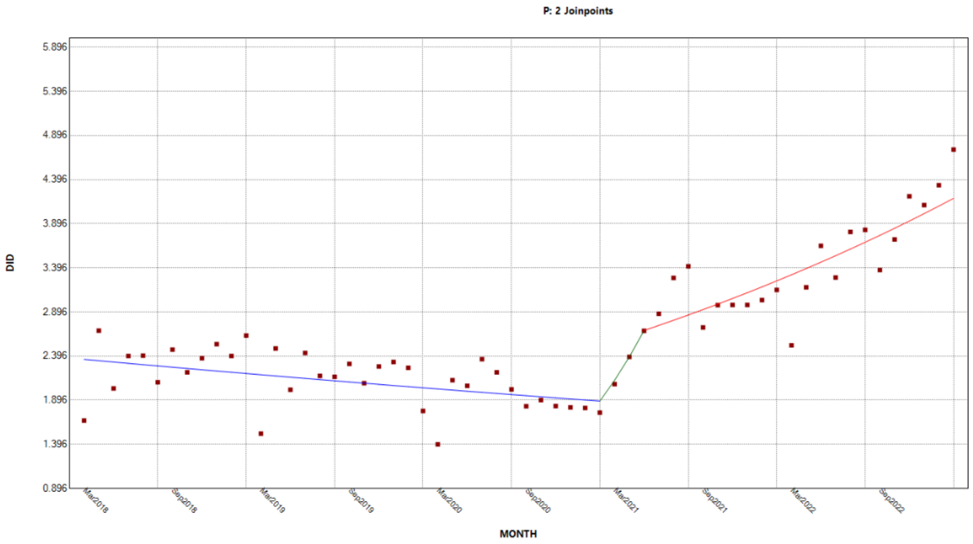

Appendix 23. Trend of utilization under insurance coverage in class P

Various (V)

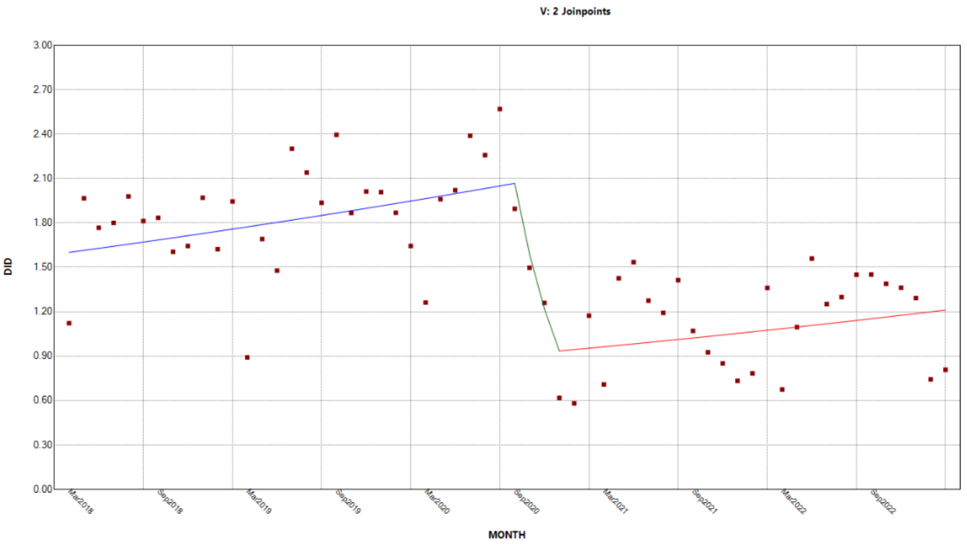

Appendix 24. Trend of utilization under insurance coverage in class V
